# High concentrations of dissolved biogenic methane associated with cyanobacterial blooms in East African lake surface water

**DOI:** 10.1038/s42003-021-02365-x

**Published:** 2021-07-07

**Authors:** Stefano Fazi, Stefano Amalfitano, Stefania Venturi, Nic Pacini, Eusebi Vazquez, Lydia A. Olaka, Franco Tassi, Simona Crognale, Peter Herzsprung, Oliver J. Lechtenfeld, Jacopo Cabassi, Francesco Capecchiacci, Simona Rossetti, Michail M. Yakimov, Orlando Vaselli, David M. Harper, Andrea Butturini

**Affiliations:** 1 Water Research Institute, National Research Council of Italy (IRSA-CNR), Monterotondo, Rome Italy; 2 Department of Earth Sciences, University of Florence, Florence, Italy; 3 Institute of Geosciences and Earth Resources, National Research Council of Italy (IGG-CNR), Florence, Italy; 4 Department of Environmental Engineering, University of Calabria, Arcavacata di Rende, Rende, Italy; 5 University of Leicester School of Geography, Geology and the Environment, Leicester, UK; 6 Department of Evolutionary Biology, Ecology and Environmental Sciences, University of Barcelona, Barcelona, Spain; 7 Department of Geology, University of Nairobi, Nairobi, Kenya; 8 Helmholtz Centre for Environmental Research (UFZ), Leipzig and Magdeburg, Germany; 9 Institute for Marine Biological Resources and Biotechnology, National Research Council of Italy (IRBIM-CNR), Messina, Italy; 10 Freshwater Biological Association, Far Sawrey, Ambleside, England UK

**Keywords:** Carbon cycle, Microbial ecology

## Abstract

The contribution of oxic methane production to greenhouse gas emissions from lakes is globally relevant, yet uncertainties remain about the levels up to which methanogenesis can counterbalance methanotrophy by leading to CH_4_ oversaturation in productive surface waters. Here, we explored the biogeochemical and microbial community variation patterns in a meromictic soda lake, in the East African Rift Valley (Kenya), showing an extraordinarily high concentration of methane in oxic waters (up to 156 µmol L^−1^). Vertical profiles of dissolved gases and their isotopic signature indicated a biogenic origin of CH_4_. A bloom of Oxyphotobacteria co-occurred with abundant hydrogenotrophic and acetoclastic methanogens, mostly found within suspended aggregates promoting the interactions between Bacteria, Cyanobacteria, and Archaea. Moreover, aggregate sedimentation appeared critical in connecting the lake compartments through biomass and organic matter transfer. Our findings provide insights into understanding how hydrogeochemical features of a meromictic soda lake, the origin of carbon sources, and the microbial community profiles, could promote methane oversaturation and production up to exceptionally high rates.

## Abstract (web summary)

Fazi et al. report on an extraordinarily high biogenic methane concentration detected in the surface water of Lake Sonachi, Kenya. Using gas chromatography and microbiome profiling, they determine that these high concentrations are associated with cyanobacterial blooms and help provide insight to methanogenesis in meromictic soda lakes.

## Introduction

Methane (CH_4_) is the second most important greenhouse gas in terms of global warming potential, reported as 28–36 times higher than that of CO_2_ over the standard 100-year period^1^. The sharp increase in atmospheric CH_4_ levels observed since 2007 has been ascribed to biomass burning, fossil fuel combustion, agricultural practices, and accelerated release from biogenic sources^2,3^, although the current observational network cannot unambiguously link recent methane variations to specific sources^4^. In particular, estimates of carbon gas fluxes across the air-water interface showed that freshwater bodies represent a source of methane with a disproportionate contribution to global CH_4_ emissions, regardless the relatively small surface they cover^5,6^.

Methane production from lakes is mainly attributed to anoxic sediments, with different emission pathways upward to the surface and the atmosphere (e.g., diffusion, ebullitive and storage fluxes, emissions from aquatic vegetation)^7^. Surface waters can be systematically oversaturated with CH_4_ through vertical and lateral transport from bottom and littoral sediments, as found in small and shallow ponds^8^. Methane oversaturation, however, was also reported in lake waters with negligible sediment-to-water exchanges, owing to known pathways of oxic methanogenesis mediated by light-, nutrient-, and salt-dependent microbial metabolisms, along with the occurrence of pelagic micro-anoxic niches^9–12^. Notably, methanogenic microorganisms were detected in association with either algae or Cyanobacteria in oxygenated epilimnion^13^ and experimental cultures of selected Cyanobacteria were likely to produce methane in saturated oxic conditions^14^. A close link between CH_4_ release and algal biomass was also confirmed by both the overlap of metalimnetic CH_4_ maxima with oxygen oversaturation and chlorophyll maxima^11,12^ and the positive relation between surface CH_4_ flux rates and chlorophyll-a levels^15–17^.

**Fig. 1:**
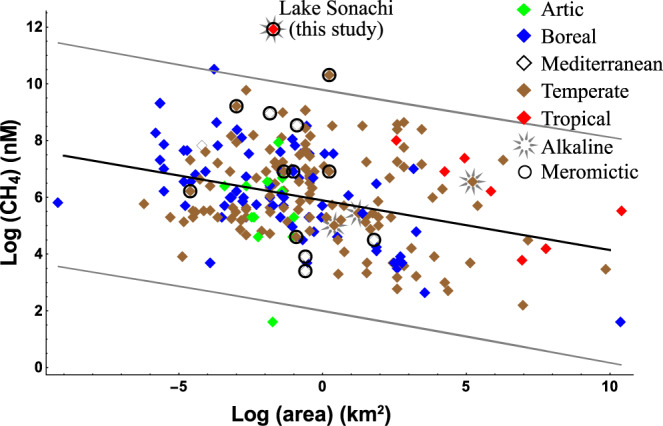
Literature overview of CH_4_ concentration in oxic lake waters. Reported values derive from lakes distributed worldwide across a range of size (i.e., area) and climatic region (i.e., arctic, boreal, Mediterranean, temperate, tropical). Alkaline and meromictic lakes are highlighted. Regression line (*p* < 0.001) and confidence interval (0.99) are reported. Extended data are reported in Supplementary Table 1.

Methane production in surface layers moves the source of CH_4_ closer to the water–air interface with a significantly higher contribution to the overall emission^9,12^, but it is not known whether, how, and to what extent methanogenic processes can counterbalance aerobic methanotrophy^18^. Therefore, consistent knowledge gaps persist on the interplays between primary production, organic matter transformation, and methane mobilization mechanisms. In the East African Rift Valley (Kenya), we discovered an unusual high concentration of methane in the oxic layer of a meromictic soda lake. The amount of dissolved CH_4_ was exceptionally higher than that reported from natural lakes across a wide range of lake size and type (Fig. 1 and Supplementary Table 1).

Saline lakes are distributed worldwide, with an estimated total volume (104 × 10^3^ km^3^) comparable to that of freshwater lakes (124 × 10^3^ km^3^)^19^. In particular, soda lakes, characterized by saline alkaline waters in which Na^+^ and carbonate species are the dominant ions, are common in regions with volcanic bedrock, including the eastern branch of the East African Rift and several lake basins across the globe^20,21^.

There is a growing debate about the relevance of tropical aquatic ecosystems in terms of methane emissions. The focus has now moved to Africa, because in situ measurements are poorly documented and frequent cloud cover reduces satellite data densities/estimates^22,23^. East African soda lakes are characterized by high salinity, high constant solar radiation, warm temperature, and high steady pH, promoting high primary production with high amount of autochthonous derived dissolved organic matter and diverse haloalkaliphilic microbial communities^19,24,25^. They are considered as model environments for a deeper understanding of microbially-driven processes, including all possible methanogenic pathways, which were found to be concurrently active up to nearly salt-saturation conditions^20,26^.

The objectives of this study were to (i) uncover the contribution of geogenic and biogenic sources to the bulk of dissolved CH_4_, (ii) identify key microbial players and interactions at different lake compartments (i.e., oxic/anoxic water layers and sediments). We tested the hypothesis that, regardless of geogenic sources, the oversaturation of methane in oxic lake waters can be promoted by microbial community structuring and cell-to-cell interactions up to unexpectedly high levels.

## Results

### Water stratification and geochemical characteristics

Lake Sonachi showed a maximum depth of 4.5 m and a pH ranging between 9.47 and 9.61. During sampling at the center of the lake, the meromictic stratification was found at −3.5 m, with a chemocline separating the mixolimnion (surface waters) from the monimolimnion (bottom waters, BW). The vertical profiles of δD-H_2_O and δ^18^O-H_2_O changed consistently below the chemocline, showing the influence of water evaporation and the lack of mixing between mixolimnion and monimolimnion (Supplementary Figs. 1 and 2). The chemocline was apparent from abrupt changes in electrical conductivity, redox potential, pH, inorganic and organic solutes, and dissolved organic matter (DOM) (Supplementary Fig. 3; Supplementary Tables 2 and 3). The redox potential (Eh_0(25 °C)_) showed high values in surface waters and an abrupt decrease in BW.

Water temperature and oxygen in the water column ranged between 20.5 and 22.4 °C, and 0.1 and 3.6 mg L^−1^, respectively. In surface waters, the vertical profiles of temperature and oxygen revealed the presence of a thermocline and oxycline at −1.5 m, discriminating between the shallow oxic surface waters (OSW) and anoxic surface waters (ASW) (Supplementary Fig. 3). Oxygen concentrations reached saturation value in OSW, when taking into account water salinity, temperature and elevation. BW were characterized by high concentrations of Na^+^, HCO_3_^−^, CO_3_^2-^ (up to 5276, 9523 and 2196 mg L^−1^, respectively), with remarkable concentrations of K^+^, Cl^−^, SO_4_^2-^, F^−^, Si and reduced sulfur species. In contrast, Ca^2+^ concentration was low (<4.64 mg L^−1^), as well as dissolved inorganic nitrogen, mainly represented by NH_4_ + NH_3_ (<0.03 mg L^−1^) (Supplementary Table 2). Dissolved organic carbon (DOC) did not change in OSW and ASW (97.3 ± 6.8 mg L^−1^) but increased significantly in BW (up to 593 mg L^−1^). The analysis of solid phase extracted DOM (SPE-DOM) showed the signal of DOM autochthonous production together with photodegradation. In particular, there was low aromaticity and high relative abundance of reduced and saturated compounds with a remarkable contribution of oxygen impoverished aliphatic-like molecules (Supplementary Fig. 3 and Supplementary Table 3).

### Vertical profiles of dissolved gases

**Fig. 2:**
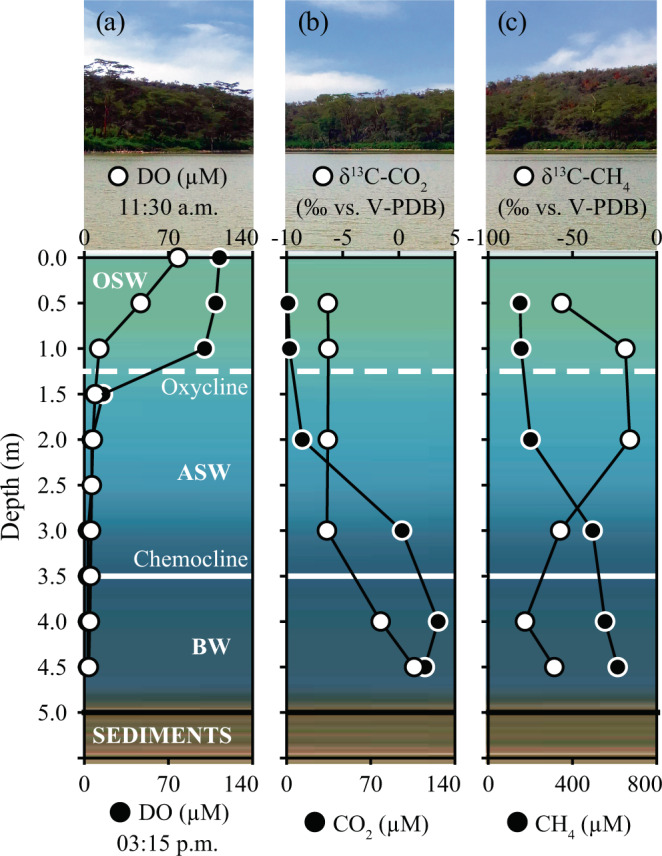
Vertical profiles of dissolved gases and isotopic signatures across the water column. **a** Concentrations of dissolved oxygen (DO, at two daytime samplings). **b** CO_2_ concentration (black dots) and δ^13^C-CO_2_ ‰ vs. V-PDB (white dots). **c** CH_4_ concentration (black dots) and δ^13^C-CH_4_ ‰ vs. V-PDB (white dots).

Inert gases did not change through the water column (Ar = 11 µmol L^−1^; He = 0.0025 µmol L^−1^). Hydrogen was only detectable in BW (0.5–0.8 µmol L^−1^). Carbon dioxide concentrations increased from the surface to the bottom, from 1 µmol L^−1^ to 115–126 µmol L^−1^. In SW, P_CO2_ was far below saturation, resulting in an estimated inward flux of atmospheric CO_2_ (up to 88.6 mg C m^−2^ d^−1^; Table 1 and Supplementary Table 4). In BW, both CO_2_ and Total Dissolved Inorganic Carbon (TDIC) were consistently enriched in ^13^C that rapidly decreased at 3 m depth (δ^13^C-CO_2_: from 1.37 to −6.41‰ vs. Vienna Pee Dee Belemnite—V-PDB—and δ^13^C-TDIC: from 10.7 to 2.91‰ vs. V-PDB) (Fig. 2 and Supplementary Table 2).

**Table 1:** Values of CO_2_ and CH_4_ fluxes (*Φ*CO_2_ and *Φ*CH_4_) estimated by applying published empirical relationships for the determination of *k*_600,i_, at *T* = 21 °C and wind speed *U*_*10*_ = 2 m s^−1^.

*k*_*600*_(cm h^−1^)	Reference	*Φ*CO_2_(mg C m^−2^ d^−1^)	*Φ*CH_4_(mg C m^−2^ d^−1^)
0.72*U*_*10*_	Crusius and Wanninkhof^73^	−46.1	591
*0.215U*_*10*_^*1.7*^*+2.07*	Cole and Caraco^88^	−88.6	1137
0.23 *U*_*10*_^*2*^+0.1 *U*_*10*_	Nightingale et al.^89^	−35.8	460

The most remarkable feature of lake waters was the exceptionally high concentration of CH_4_ in OSW. The highest level was measured at BW (615 µmol CH_4_ L^−1^), which decreased rapidly to 201 µmol L^−1^ in ASW. This decrease stopped abruptly at OSW, where CH_4_ stabilized at 151–156 µmol L^−1^, leading to an estimated water-air CH_4_ diffusive flux between 460 and 1137 mg C m^−2^ d^−1^ (Table 1). The δ^13^C-CH_4_ values ranged from −78 in BW to −16 in SW ‰ vs. V-PDB (Fig. 2).

**Fig. 3:**
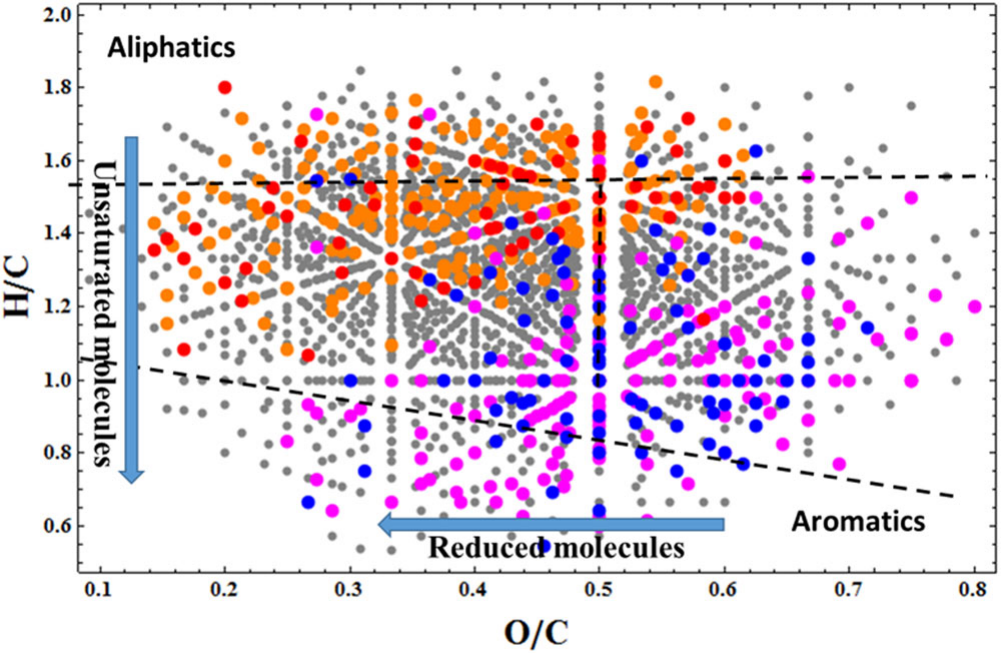
Van Krevelen diagram of all SPE-DOM molecules identified with FT-ICR MS. The diagram cross-plots the hydrogen:carbon atomic ratio (H/C) as a function of the oxygen:carbon atomic ratio (O/C). The significance of Spearman correlation between relative abundances of molecules and methane concentration is indicated by the different colors, as follows: Gray = No significance (*p* > 0.01); Red = positive significant correlation (*p* < 0.005); Orange = positive significant correlation (0.005 < *p* < 0.01); Blue = negative significant correlation (*p* < 0.005); Magenta = negative significant correlation (0.005 < *p* < 0.01).

SPE-DOM analysis showed that 27.5% of all detected aliphatic-like molecules covaried together with CH_4_. On the contrary, 34% of the assigned aromatic-like ones were inversely related to CH_4_ concentration (Fig. 3, Supplementary Table 5).

### Microbiome profiling

**Fig. 4:**
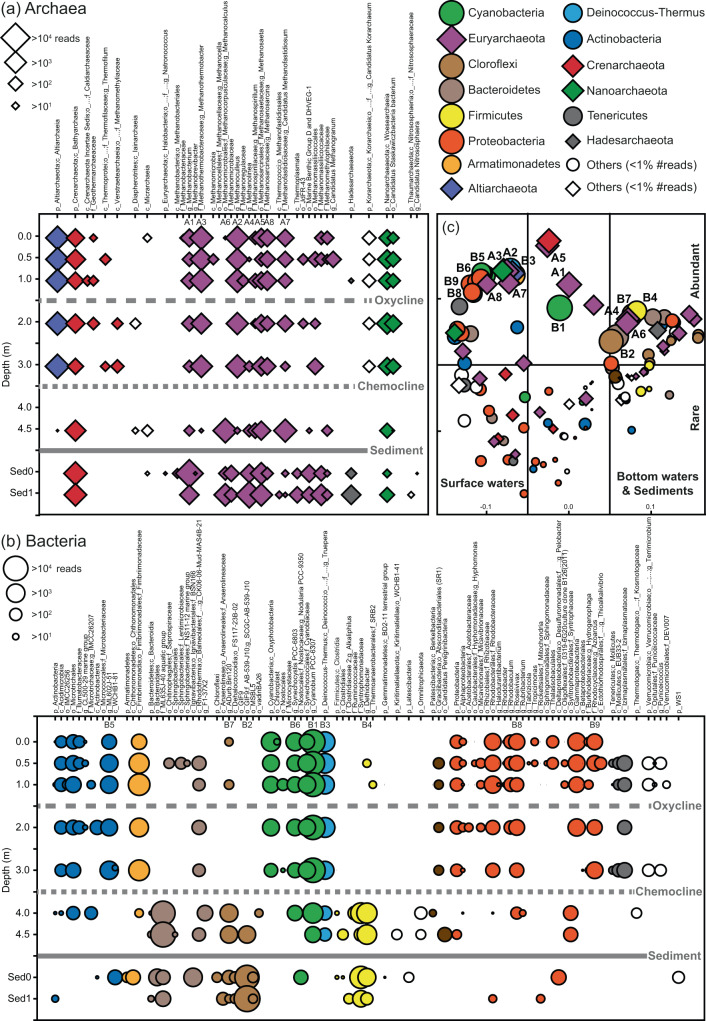
Microbial community composition across the water column and sediment of Lake Sonachi. All retrieved taxa of **a** Archaea and **b** Bacteria are represented with symbol size proportional to the number of Illumina-sequencing reads. Dominant genera are ordered by letters and numbers (Archaea = A1, A2, A3, etc.; Bacteria = B1, B2, B3, etc.). **c** The PCoA ordination plot, based on Bray–Curtis similarity index, was used to graphically visualize preferential associations among taxa and their relative distribution within the lake.

In surface waters, the archaeal community was dominated by members of the classes Altiarchaeia, Methanobacteria (genera *Methanobacterium*, *Methanothermobacter*), and Methanomicrobia (genera *Methanoculleus*, *Methanosaeta* and *Methanosarcina*) (Fig. 4a and Supplementary Table 6). Remarkably, a fraction of archaeal Amplicon Sequence Variance (ASV) at 2–3 m depth was not identified using known databases (8–11% of total reads). Cyanobacteria of the class Oxyphotobacteria were identified as the most abundant lineage (on average 60% of total reads), mainly represented by the genus *Cyanobium PCC-6307* (Fig. 4b and Supplementary Table 7). In particular, among the four main ASVs belonging to *Cyanobium* (ASV4-7) found in the mixolimnion, ASV7 reached up to 27% of total reads. Other Cyanobacteria belonged to *Synechocystis PCC-6803* (ASV1-3) with relative abundance reaching up to 4% of total reads (Supplementary Table 8). Fimbriimonadia (family Fimbriimonadaceae), Deinococci (only represented by the genus *Truepera*), and Actinobacteria (genus ML602J-51) were relatively abundant. Proteobacteria were not abundant and mainly represented by Alphaproteobacteria (genus *Roseivivax*) and Gammaproteobacteria (genus *Azoarcus*) (Fig. 4b and Supplementary Table 7).

In BW, Bacteroidia of the family ML635J-40 aquatic group reached a relative abundance higher than 80% of total reads. The euryarchaeotal Thermococci of the family Methanofastidiosaceae (genera *Candidatus Methanofastidiosum*) represented the second most abundant group (>10%).

In sediments, the euryarchaeotal genera *Methanobacterium*, *Methanolinea*, *Methanosaeta* and *Methanocalculus* dominated the community along with members of the classes Bathyarchaeia and Thermoplasmata. The sediment bacterial community showed high dominance of Chloroflexi, mainly represented by Dehalococcoidia of the genus *SCGC-AB-539-J10* in subsurface sediments (up to 93.0% of total reads). Clostridia (genus *Dethiobacter*) showed high percentages in surface sediments (28.2%) (Fig. 4a, b and Supplementary Tables 6 and 7).

The microbial communities retrieved above and below the chemocline were consistently different in terms of phylogenetic structure (one-way PERMANOVA, Bray-Curtis similarity index, *p* = 0.016). No statistical differences were found either between OSW and ASW (*p* = 0.59) or between BW and sediments (*p* = 0.33). The Principal Coordinate Analysis (PCoA) ordination plot allowed the relatively closer associations among all identified taxa of Bacteria and Archaea to be visualized (Fig. 4c).

### Quantitative assessment of microbial community structure

**Fig. 5:**
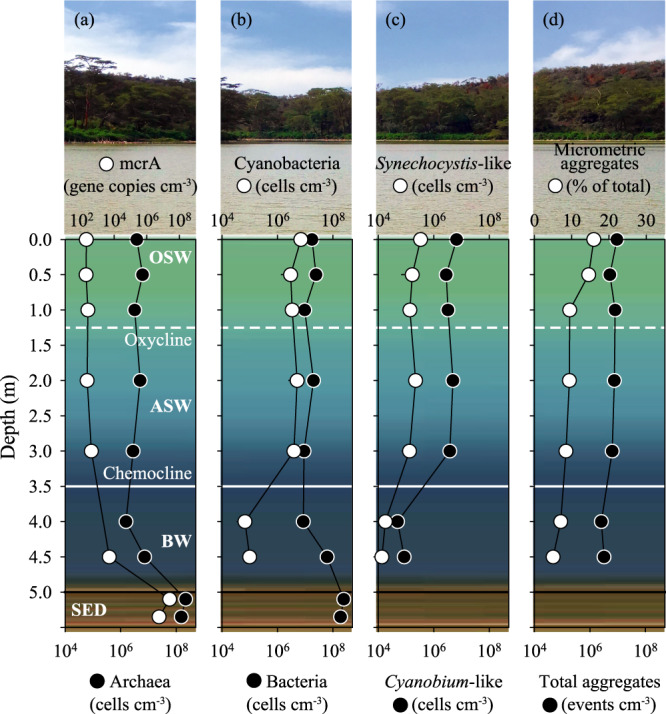
Vertical profiles of microbial community across the water column. **a** Abundance of Archaea and *mcrA* gene copies. **b** Abundance of Bacteria and Cyanobacteria. **c** Abundance of *Cyanobium*-like and *Synechocystis*-like cells. **d** Concentration of total aggregates and percentage of micrometric aggregates (>1 μm). Error bars for the microscopy observations (*n* = 3) are within the size of the symbols.

The Chlorophyll-a (Chl-a) signal concentration ranged from 93.6 ± 5.5 µg L^−1^ in surface waters to 28.5 ± 12.0 µg L^−1^ in BW. The qPCR assays revealed a high abundance of genes involved in the CH_4_ production pathway in both the water column and sediments. In particular, abundance of the *mcrA* gene in the water column increased with increasing depth, showing the lowest value at 0.5 m (190 ± 42 gene copies cm^−3^) and the highest at 4.5 m (5.1 × 10^3^ ± 1.0×10^3^ gene copies cm^−3^). The *mcrA* gene was highly abundant in sediments with values ranging between 5.0 × 10^6^ ± 3.7×10^5^ and 2.2 × 10^7^ ± 1.0 × 10^3^ gene copies cm^−3^ (Fig. 5).

Bacteria and Archaea represented respectively 72.3 ± 9.0% and 17.6 ± 6.6% of the total DAPI-stained cells in the water column. The sediments showed percentages of 53.9 ± 1.6% and 46.1 ± 1.6% for Bacteria and Archaea, respectively. *Cyanobium*-like cells represented on average 91.1 ± 8.6% of total Cyanobacteria. The majority of the microbial biomass in water was part of the particulate OM (on average >77% of total cell counts), as assessed by flow cytometry comparing unfiltered and GFF-filtered (0.7 µm pore size) aliquots. In particular, >99% of the total autofluorescent pigmented cells were removed by GFF filtration. The concentration of total microbial aggregates was in the range of 2.6 × 10^6^ to 9.4 × 10^6^ aggregates cm^−3^ and decreased with depth, along with the percentage of micrometric aggregates (Fig. 5).

**Fig. 6:**
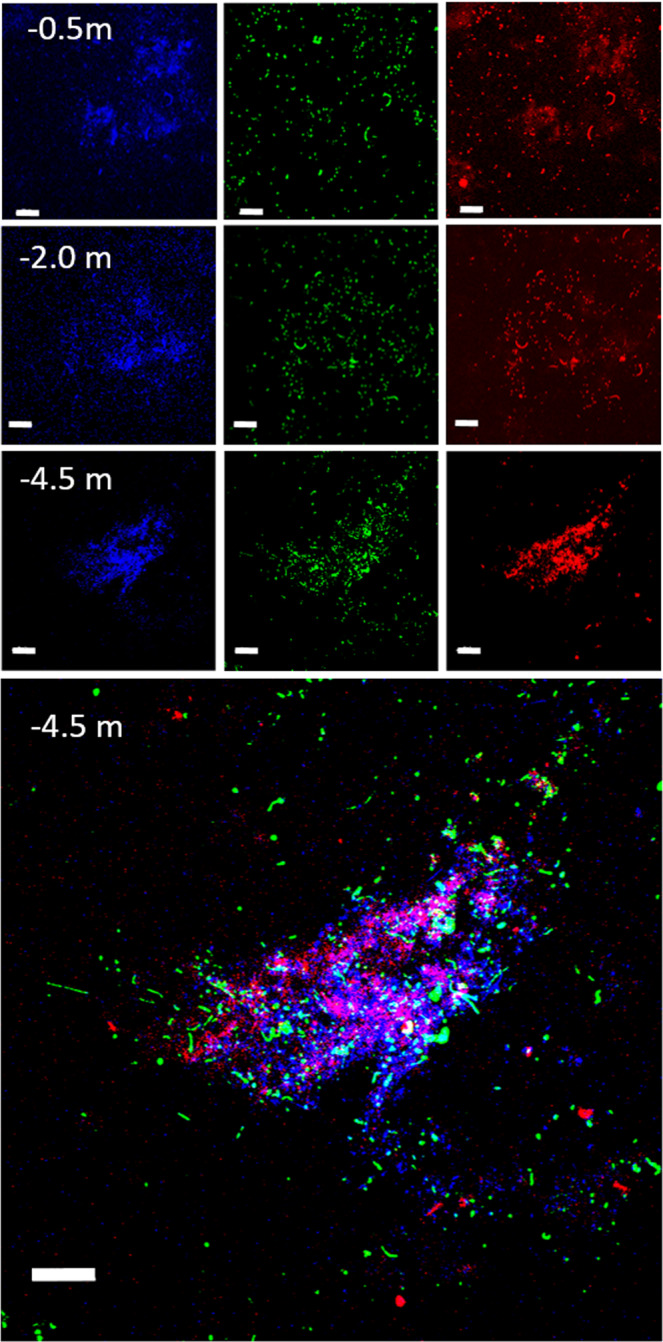
CLSM combined images showing microbial aggregates at 3 different depths. DAPI stained cells (blu), bacterial cells identified by CARD-FISH (green); autofluorescent Cyanobacteria (red). Lowest panel shows the overlap of three images (Bars = 20 μm).

The visual inspection by confocal microscopy confirmed the occurrence of microbial cells clustered in micrometric aggregates (Fig. 6). A close association between Archaea and Bacteria, including Cyanobacteria, was visualized within suspended microbial aggregates by epifluorescence and confocal microscopy (Supplementary Fig. 4).

## Discussion

This study reports the accumulation of an unusual high amount of biogenic methane in surface oxic waters of the meromictic soda lake Sonachi, occurring together with a high availability of autochthonous dissolved organic matter and abundant cyanobacteria-bacteria-methanogens interacting cells. The amount of dissolved CH_4_ was 1–3 orders of magnitude higher than that reported in oxic layers of other natural lakes, regardless of their geochemical setting, morphology (e.g., area, depth), and trophic status (e.g., Chl-a, DOC) (Fig. 1, Supplementary Fig. 5, Supplementary Table 1). The measured values were comparable only to those reported from high latitude lakes in winter when CH_4_ released from sediments is trapped at the water–ice interface^27^ or during hypolimnion overturn episodes^28^.

Dissolved CH_4_ concentrations were exceptionally high in OSW and corresponded to an estimated water-to-air CH_4_ flux of up to 1137 mg C m^−2^ d^−1^ (Table 1). Recent findings have highlighted regional hot-spot methane emissions in South Sudan (the Sudd swamp), Southern Africa (wetlands in Zambia, Angola and Botswana), Congo (floodplains of the River Congo), and around lakes Victoria, Kyoga and Albert^22,29^. Our study has provided evidence that highly productive soda lakes from the East African Rift might also be remarkable CH_4_ sources in tropical settings. The water-to-air CH_4_ flux, calculated according to the thin boundary layer model from dissolved CH_4_ concentration, appeared to be among the largest diffusive estimates from a lake, with values of the same order of those reported for rice fields^30^, the Amazon floodplain^31^, and wetlands^32^.

It is worth noting that our estimation is probably a conservative approximation because direct flux measurements of CH_4_ ebullition were not performed in this study. The formation of visible bubbles was not observed and the measured total dissolved gas pressure did not exceed hydrostatic pressure along the depth profile (Supplementary Table 4). However, bubbling from sediments and bottom waters might be triggered by system perturbations (e.g., promoting sediment resuspension), temperature increase (resulting in lowered CH_4_ solubility and increased methanogenesis^33^), and water level decline (resulting in decreased hydrostatic pressure), which could follow possible changes in the balance between rainfall and evaporation within the endorheic basin of Lake Sonachi.

The vertical profile of CH_4_ showed a sharp decrease approaching the surface, indicating the prevalence of methane oxidation processes, as confirmed by the strong ^13^C enrichment in ASW. With this rate of decrease, CH_4_ should become depleted at an approximate depth of 1.2 m. However, the decrease in δ^13^C-CH_4_ values observed in OSW suggested the occurrence of an additional source of CH_4_ likely balancing methanotrophy. Previous studies reporting ^13^C depletion in oxic waters from other systems similarly invoked the occurrence of oxic CH_4_ production^12,34^. Notably, the ^13^C depletion reported by these studies was about half of the isotopic variation found in OSW^12,34^, thus indicating an outstanding CH_4_ production in oxic conditions in Lake Sonachi. Although lateral transport from the littoral zone has been similarly invoked to explain CH_4_ supersaturation in surface water^28,29^, such hypothesis seemed to be unlikely in the case of the endorheic crater Lake Sonachi, where horizontal water movements were largely hindered by (i) the conic lake morphology that reduces the action of winds and (ii) the lack of in- and out-flowing waters.

The high CH_4_ concentration occurred in a context of unlimited availability of inorganic carbon, high DOC and Chl-a values, high and steady temperatures. DOC was rich in oxygen-poor saturated-like compounds, thus reflecting the autochthonous microbial origin together with photodegradation^25^. Literature reports showed that labile autochthonous phytoplanktonic OM enhanced methane production in freshwater lake sediments^35,36^. Moreover, DOM photooxidation can release molecules acting as electron acceptors and carbon sources in CH_4_ production^37^.

High CH_4_ concentrations in oxic waters were related to chlorophyll peaks and current observations suggest a link between planktonic primary producers and methanogens, putatively mediated by DOM release from pelagic microbial primary producers^13,17^.

Concomitantly, Lake Sonachi was also a remarkable net CO_2_ sink, with a CO_2_ uptake rate comparable to those reported for other eutrophic lakes in temperate areas^38,39^. The CO_2_ inward flux reflected high pH and chlorophyll concentration (high primary productivity in the mixolimnion). In an earlier study spanning 15 months, Melack^40^ reported a net daily oxygen production exceeding the nightly oxygen consumption (respiration) in six out of nine cases, and concluded that Lake Sonachi should be a net CO_2_ sink during most of the year. Here, the δ^13^C-TDIC value measured in bottom waters (up to 10.7‰ vs. V-PDB) was in line with that reported elsewhere^41^, one of the highest δ^13^C-TDIC values reported for natural lakes, to the best of our knowledge. These high values pointed to biomass-dependent carbon fractionation through CO_2_ fixation by chemosynthetic organisms and CO_2_ consumption due to methanogenesis, also observed in pore waters from other alkaline lakes from East Africa^42^. The process strongly affected the isotopic composition of CO_2_ in bottom waters. Thus, the possible occurrence of CO_2_ from mantle/magmatic degassing and/or from carbonate-rich sediments^43^ cannot be recognized by the isotopic signature of CO_2_, clearly excluding the input of geogenic gases. Moreover, CO_2_ migrating upward due to diffusion is affected by other consumption processes related to photosynthesis and dissolution as carbonate ions. Both these processes caused a ^13^C increase in the residual CO_2_, which was counteracted by the production of ^12^C-rich CO_2_ from CH_4_ oxidation. Such a complex superimposition of processes may explain the vertical profile of the δ^13^C-CO_2_ values (Fig. 2).

**Fig. 7:**
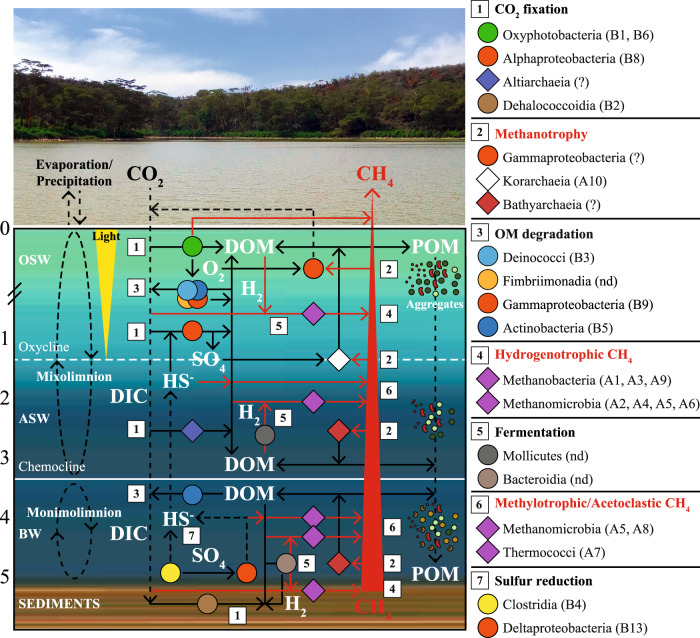
Conceptual model of major C-cycle related processes. Box 1-7 processes mediated by key abundant archaeal and bacterial classes (diamonds and circles) as detected by amplicon sequencing in samples from Lake Sonachi. Dominant genera are ordered by letters and numbers (Archaea = A1, A2, A3, etc.; Bacteria = B1, B2, B3, etc.). Solid red arrows refer to processes directly involved in methane metabolism. Solid red arrows refer to processes involving dissolved solutes. Dashed black arrows refer to crossing-chemocline release of gases (e.g., CO_2_, H_2_S) and sedimentation of POM and microbial aggregates. Functional assignments are based on putative metabolic activities of known prokaryotic taxa retrieved at the biogeochemical conditions across the lake section.

By combining hydrogeochemical features, the origin of carbon sources, and microbial community profiles, we developed a conceptual model of major C-cycle related processes, as mediated by key microbial taxa (Fig. 7). In OSW, the abundance and identity of methanogenic Archaea, along with the occurrence of *mcrA* gene, provided evidence of microbial methanogenesis (Fig. 7, box 4) in waters with high primary production (Fig. 7, box 1). The genera *Methanobacterium*, *Methanoculleus*, and *Methanothermobacter* were the most abundant putative hydrogenotrophs, as most of the known members of Methanobacteria and Methanomicrobia^44^. Notably, the acetoclastic methanogen *Methanosaeta* co-occurred with fermentative *Izimaplasmataceae*, as also reported elsewhere^45^. Members of *Methanofastidiosacea*, detected in all samples, could also contribute to CH_4_ production through the H_2_-utilizing methylotrophic pathway^46,47^. The highly methane productive system was likely fed by carbon fixation, mediated by well-known photosynthetic Cyanobacteria (i.e., *Cyanobium* PCC-6307 and *Synechocystis* PCC-6803) and anoxigenic photosynthetic bacteria (e.g., *Rhodobacteraceae*) in the illuminated surface waters. CO_2_ fixation could be also carried out by members of the class Altiarchaeia. Representatives of the ‘*Candidatus Altiarchaeum hamiconexum*’ were found as dominant primary producers in anaerobic environmental conditions in which CO_2_ fixation can be mediated by a novel variant of Acetyl-CoA pathway^48^. The high abundance of Cyanobacteria across the water column may suggest a direct involvement of photosynthetic bacteria in CH_4_ production. This is possibly due to cellular release of precursors of methylated compounds produced to cope with high salinity. It is worth noticing that *Synechocystis* PCC-6803 was reported to contain only the bidirectional hydrogenase that seems insensitive to oxygen^49^. Moreover, there is emerging evidence that some Cyanobacteria may directly produce CH_4_ by demethylation, completely bypassing the involvement of heterotrophic microorganisms. Moreover, members of Bathyarchaeota could contribute to methanogenesis but, by means of the reversible Mcr complex, they could also mediate methanotrophic processes^50^. Bathyarchaeota are reported to form the backbone of the archaeal community, often co-occurring with Methanomicrobia^51^. Methanotrophic pathways could additionally be linked to dissimilatory sulfur reduction through the sulfide-dependent anaerobic oxidation of CH_4_ to methanol mediated by members of Korarchaeota^52^, herein retrieved only in surface waters (Fig. 7, box 2).

In BW and sediments, methanogenesis was mainly driven by acetoclasts (i.e., Methanosaeta), although hydrogenotrophs were also retrieved at high relative abundance (i.e., members of genera *Methanobacterium*, *Methanolinea*, and *Methanocalculus*) (Fig. 7, box 6). In line with previous findings from hypersaline soda lakes^53^, our results suggested that hydrogenotrophic, acetoclastic, methylotrophic, and H_2_-dependent methylotrophic methanogenic pathways can all be energetically favorable at haloalkaline conditions. CO_2_ fixation was likely linked to acetogenesis mediated by Cloroflexi of the genus SCGC-AB-539-J10 through the reductive acetyl-CoA pathway^54,55^ (Fig. 7, box 1). Unclassified Chloroflexi were found to be involved in acetogenesis in mofette soil^56^.

In addition, the occurrence of heterotrophic and fermentative bacterial taxa along the water column confirmed that lake functioning was fundamentally based on OM degradation processes (Fig. 7, box 3, 5). Notably, fermentation was putatively mediated by Bacteroidetes of the genus ML635J-40 aquatic group. In anaerobic reactors setup with sediment from a soda lake, the supplied *Spirulina*-derived substrate was mainly hydrolyzed by Bacteroidetes from the ML635J-40 aquatic group^57^.

The high relative abundance of sulfur (non-sulfate) reducing bacteria (i.e., *Dethiobacter* spp.) suggested that high salinity could prevent sulfate reduction, thus lowering competition for H_2_ and organic substrates with methanogens (Fig. 7, box 7).

Phenotypic characteristics and structural patterns of the microbial community can play a fundamental role in lake functioning, in addition to the phylogenetic diversity with putative functional assignments found here. Microbial communities can show heterogeneous behavior while adapting to a changing environment to optimize resource utilization, even when cells are genetically identical^58,59^. In particular, the dynamics of microbial aggregates could provide underinvestigated clues supporting methanogenesis in oxic waters. Cell density and proximity could lead to direct interactions among Bacteria, Cyanobacteria, and Archaea, promoting methane production^13,60^. Aggregate settling can also increase OM availability in bottom waters and sediments, since clustered cells can move across the chemocline more rapidly than single cells^61^. The breakdown of settled aggregates, induced by abrupt water chemistry changes, could accelerate the release of intracellular methylated compounds, further supporting methanogenesis below the chemocline through fermentative and acetogenic processes.

In conclusion, our findings provide insights towards understanding how hydrogeochemical features, the origin of carbon sources, and microbial community profiles could lead to an exceptionally high concentration of dissolved biogenic methane in a meromictic soda lake. As lake functioning is influenced by water stratification and primary production under oxic and anoxic conditions, both genotypic and phenotypic microbial community changes can affect methane fluxes, with direct yet overlooked consequences for greenhouse gas emissions and climate feedbacks under accelerating global trends of lake eutrophication.

## Material and methods

### Study site

Lake Sonachi (meaning ‘barren bull’ from Masaai and previously referred to as Crater Lake) is a endorheic meromictic volcanic soda lake, located at about 90 km NW of Nairobi at 1884 m a.s.l., within the Eastern Rift Valley in central Kenya to the immediate South West of the freshwater Lake Naivasha (Supplementary Fig. 6). The lake surface area is around 0.18 km^2^, with a maximum depth of ~5 m. Local climate is warm and semiarid, with evaporation exceeding precipitation on an annual basis. Protection from wind by steep crater walls (rising up from 30 to 115 m above the lake surface) and vegetation (mainly *Vachellia xanthophloea*) limit water mixing. The hydrological balance is maintained by precipitation (~680 mm/year in the crater catchment) and evaporation (~1870 mm/year). Furthermore, the occurrence of subsurface inflow from the nearby Lake Naivasha was proposed according to synchronous lake-level changes among the two lakes and other hydrological evidences. Chemical stratification and meromixis were documented across 8 years of periodic measurements and attributed to several local factors, including basin morphometry, diurnal periodicity of winds and thermal stratification, seasonal/yearly rainfall variations, and biological decomposition^62,63^.

### Sampling procedures and field measurements

Water temperature, pH, electrical conductivity, dissolved O_2_ and Oxidation Reduction Potential were measured by means of multiparameter YSI sensors at regular depth intervals of 0.5 m along one single vertical profile in the deepest part of the lake, immediately before and after sampling (i.e., at 11.30 a.m. and 3.15 p.m.). Water and dissolved gas sampling was carried out down a vertical profile from surface to bottom (0, 0.5, 1, 2, 3, 4, and 4.5 m depth) using the single hose method^64^. Two unfiltered aliquots were collected in 125 mL polyethylene bottles for the analysis of major anions and stable isotopes. Two filtered (0.45 µm) and acidified (0.5 mL ultrapure HCl and HNO_3_, respectively) aliquots were collected in 50 mL polyethylene bottles for the analysis of major cations and trace species, respectively. For the analysis of total reduced sulfur species (ΣS^2−^), 8 mL of unfiltered water were collected in 15 mL plastic tubes after the addition of 2 mL of a Cd-NH_3_ solution^65^. For the isotope analysis of Total Dissolved Inorganic Carbon (TDIC), 6 mL of unfiltered water were collected in a pre-evacuated 12 mL glass vial filled with 2 mL H_3_PO_4_ and equipped with a pierceable septum. Dissolved gases were sampled in a pre-evacuated 250 mL glass flask, equipped with a Teflon stopcock, connected to the Rilsan^®^ tube^64^. For DOM analysis, 50 mL filtered (combusted GFF Whatman filters) and acidified (HCl) aliquots were stored in pre-combusted glass bottles. For the analysis of microbial diversity, lake water (500 mL) was collected at each depth, filtered with 0.2 µm pore-size polycarbonate filters (type GTTP; diameter, 47 mm; Millipore, Eschborn, Germany) and stored at −20 °C. For the analysis of community composition by CARD-FISH, a further aliquot (15 mL) was fixed with a formaldehyde solution (Sigma Aldrich; final concentration 1%). Sub-aliquots of 5–10 mL were filtered at low vacuum levels (<0.2 bar) onto 0.2 µm pore-size polycarbonate filters. Filters were stored at −20 °C until further processing. Unfiltered and (GFF Whatman) filtered (2 mL) aliquots were fixed as above and stored at 4 °C for cytometric analysis. Sediments collected by a grab from the bottom of the lake were divided into sub-aliquots, either (i) directly stored at −20 °C (5 mL), or (ii) fixed with ethanol (Sigma Aldrich; final concentration 50%) and stored at −20 °C (50 mL) until further processing.

### Chemical and isotopic analyses of water samples

HCO_3_^−^ and CO_3_^2−^ were analyzed by acidimetric titration (HCl 0.01 N). Main dissolved anions (Cl^−^, SO_4_^2−^, Br^−^, F^−^) and cations (Na^+^, K^+^, Ca^2+^, Mg^2+^) were analyzed by ion chromatography using Metrohm 761 and Metrohm 861 chromatographs, respectively (analytical error <5%). Water samples (100 mL) were filtered (0.45-µm pore-size nylon filters) and acidified with ultrapure HCl for dissolved inorganic nitrogen (DIN = NH_4_^+^ + NH_3_ + NO_2_^−^ + NO_3_^−^) determination. NO_2_^−^ and NO_3_^−^ were assessed using a colorimetric method on Bran+Luebbe autoanalyzer after nitrate reduction in a copper-cadmium column. NH_4_^+^ + NH_3_ concentrations were estimated by the salicylate method^66^. To remove silica interference, the pH of the solution was set to 1. To reduce interference by salts and sulfite, all samples were previously diluted to 1/10 −1/30. Phosphate was determined using the molybdate method^67^.

Trace elements were analyzed by inductively coupled plasma optical emission spectrometry using a PerkinElmer Optima 8000 (analytical error <10%). Reduced sulfur species were analyzed as SO_4_^2−^ after oxidation with H_2_O_2_ of CdS resulting from the reaction of ΣS^2−^ with the Cd-NH_3_ solution^65^. ^18^O/^16^O and D/^1^H isotopic ratios of water (expressed as δ^18^O-H_2_O and δD-H_2_O in ‰ vs. V-SMOW) and ^13^C/^12^C isotopic ratio of TDIC (expressed as δ^13^C-TDIC in ‰ vs. V-PDB) were analyzed using a Finnigan Delta Plus XL mass spectrometer^64^. Analytical errors for δ^18^O-H_2_O, δD-H_2_O, and δ^13^C-TDIC were ±1‰, ±0.1‰, and ±0.05‰, respectively. Unfiltered aliquots were also employed to quantify underwater light attenuation by performing light absorption spectra between 280 and 1000 nm.

### DOM characterization

DOC and dissolved organic nitrogen were determined by oxidative combustion and IR analysis using a Shimadzu total organic carbon analyzer coupled to a Total Nitrogen unit. An elemental formula data set from Fourier transform ion cyclotron resonance mass spectrometry was available and reported earlier for the same sampling campaign^25^. The inter sample ranks (for components which were present in all seven depths) which were calculated in that study were used for the calculation of the Spearman’s rank correlation with the methane concentrations. The calculation of the rank correlation coefficients and the assignment of levels of significance to elemental formula components and its visualization in van Krevelen diagrams were described elsewhere^68^.

### Gas composition

The chemical composition of dissolved inorganic gases in the headspace of the sampling flask (CO_2_, N_2_, Ar, H_2_, He) was determined using a Shimadzu 15A gas chromatograph equipped with a Thermal Conductivity Detector, whereas CH_4_ was analyzed using a Shimadzu 14A gas chromatograph equipped with a Flame Ionization Detector. Instrument specifications and analytical procedures were described elsewhere^64^. The analytical error of the GC analysis was ≤5 %. Assuming that gases in the headspace of the sampling flasks were in equilibrium with the liquid, the number of moles of each gas species in the liquid (n_i_l) was computed on the basis of those measured in the flask headspace (n_i_g) by means of the Henry’s law constants^69^. The total moles of each dissolved gas species (n_i_t) was given by the sum of n_i_l and n_i_g. The partial pressures of each gas species were computed based on the total mole values according to the ideal gas law. The isotopic composition of CH_4_ (expressed as δ^13^C-CH_4_ in ‰ vs. V-PDB) collected in the headspace of the sampling flask was analyzed by Wavelength-Scanned Cavity Ring Down Spectroscopy (WS-CRDS) using a Picarro G2201-i analyzer. The isotopic composition of dissolved CO_2_ (expressed as δ^13^C-CO_2_ in ‰ vs. V-PDB) was calculated from measured δ^13^C-TDIC assuming the attainment of chemical and isotopic equilibria among dissolved carbon species, as follows (Eq. (1)):

1 $${\partial }^{13}{C}_{{CO}2}	=\; {\partial }^{13}{C}_{{TDIC}}-\left(\frac{{{HCO}}_{3}}{{TDIC}}\times {\epsilon }_{{HCO}3-{CO}2}\right)\\ 	\quad-\left[\frac{{{CO}}_{3}}{{TDIC}}\times \left({{\epsilon }_{{CO}3-{HCO}3}+\epsilon }_{{HCO}3-{CO}2}\right)\right]$$

where HCO_3_^−^, CO_3_^2−^, CO_2_, and TDIC concentrations were expressed in mmol L^−1^; the equilibrium isotopic enrichment factors ε_HCO3-CO2_ and ε_CO3-HCO3_ were calculated according to previous methods^69,70^, as follows (Eqs. (2) and (3)):

2 $${\epsilon }_{{HCO}3-{CO}2}=\frac{9866}{T}-24.12$$

3 $${\epsilon }_{{CO}3-{HCO}3}=1.85-\frac{666}{T}$$

where *T* is temperature in degrees Kelvin.

### Water–air CO_2_ and CH_4_ diffusive fluxes

The water–air CO_2_ and CH_4_ diffusive exchange fluxes (*Φ*CO_2_ and *Φ*CH_4_, respectively) were calculated, according to the thin boundary layer (TBL) model^71^, from dissolved gas concentrations measured in surface water (0.5 m depth) and gas transfer velocities (*k*_*i*_, in cm h^−1^), as follows (Eq. (4)):

4 $${\Phi }_{{\rm{i}}}={\beta k}_{i}\big({C}_{i,w}-{C}_{i,{eq}}\big)$$

where *C*_*i,w*_ was the dissolved gas concentration measured in surface water (in mol L^−1^), *C*_*i,eq*_ was the dissolved gas concentration calculated assuming equilibrium with the atmosphere (based on Bunsen coefficients compiled by Wanninkhof^72^ as a function of temperature and salinity), and β was the chemical enhancement applicable for CO_2_ only (see below). The *k*_*i*_ values were estimated as follows (Eq. (5)):

5 $${k}_{i}={k}_{600,i}{\left(\frac{{{Sc}}_{i}}{600}\right)}^{x}$$

where *Sc*_*i*_ was the Schmidt number (i.e., the ratio of kinematic viscosity of water and the diffusion coefficient of the gas), *k*_*600,i*_ was the transfer coefficient for each gas normalized to 600, and the power dependence *x* was dependent upon the roughness of the water surface (−0.67 or −0.5 for wind speed <3 m s^−1^ or >3 m s^−1^, respectively;^73^). *Sc*_*i*_ and *k*_*600,i*_ values are specific for each gas species and depend on temperature and wind speed, respectively. The *Sc*_*i*_ values were determined using the fourth-order polynomial fit proposed by Wanninkhof^72^ (Eqs. (6) and (7)):

6 $${{Sc}}_{{{CO}}_{2}}=2116.8-136.25T+4.7353{T}^{2}-0.09231{T}^{3}+0.0007555{T}^{4}$$

7 $${{Sc}}_{{{CH}}_{4}}=2101.2-131.54T+4.4931{T}^{2}-0.08676{T}^{3}+0.00070663{T}^{4}$$

where *T* was the temperature in °C.

The *k*_*600,i*_ values were calculated from local wind speed using the empirical relationships reported in Table 1, where *T* was the temperature measured in surface water (⁓21 °C) and *U*_*10*_ was the wind speed at a height of 10 m. During sampling, wind speed was low, but the exact velocity was not measured. Nevertheless, according to Melack^40^ and Melack and MacIntyre^74^, wind speeds at ~2 m above water surface at Lake Sonachi were frequently low (<2 m s^−1^) and averaged 2–4 m s^−1^, with gusts only occasionally exceeding 6 m s^−1^. Consequently, an average *U*_*10*_ value of 2 m s^−1^ was adopted for estimating gas diffusive fluxes.

Since pH in Lake Sonachi was ≥9.5, CO_2_ was expected to undergo hydration and hydroxylation reactions (i.e., ${{CO}}_{2}+{H}_{2}O={H}_{2}{{CO}}_{3}$; ${{CO}}_{2}+{{OH}}^{-}={H{{CO}}_{3}}^{-}$), augmenting the flux of atmospheric CO_2_ into the lake (chemical enhanced diffusion). The chemical enhancement factor β was computed according to the model proposed by Hoover and Berkshire^75^ and Wanninkhof and Knox^76^, as follows (Eq. (8)):

8 $$\beta =\frac{\tau}{({{\tau }}-1)+\tan h\left[{(r{{\tau }}{{\rm{D}}}^{-1})}^{0.5}D{k}_{{{CO}}_{2}}^{-1}\right]/ \left[{(r{{\tau }}{{\rm{D}}}^{-1})}^{0.5}D{k}_{{{CO}}_{2}}^{-1}\right]}$$

where: (i) *D* was the molecular diffusivity (in cm^2^/s), calculated according to Zeebe^77^, i.e., $D=14.6836\times 10^{-5}\times [(273.15+t({\,}{\!}^{\circ} C))/217.2056-1]^{1.997}$; (ii) *r* (in s^−1^) was the combined rate constant for the hydration of CO_2_ either directly or via carbonic acid, calculated as $r={r}_{1}+{r}_{2}{K}_{w}^{\ast }{a}_{H}^{-1}$, where *r*_*1*_ (in s^−1^) and *r*_*2*_ (in L mol^−1^ s^−1^) were the CO_2_ hydration rate constant and the CO_2_ hydroxylation rate constant, respectively^78^, *K*_w_^*^ was the equilibrium constant for water, and *a*_*H*_ was the activity coefficient for the hydrogen ion; (iii) ${\rm{\tau }}=1+{a}_{{\rm{H}}}^{2}/\left({{\rm{K}}^{\prime} }_{1}{{\rm{K}}^{\prime} }_{2}+{{\rm{K}}^{\prime} }_{1}{a}_{{\rm{H}}}\right)$, where K’_1_ and K’_2_ were the first and second equilibrium constants for carbonic acid, respectively^79^.

### High-throughput 16S rRNA amplicon sequencing and bioinformatics

Extracted DNA was amplified in a first PCR with the primer pairs 27F (5′-AGAGTTTGATCCTGGCTCAG-3′) and 534R (5′-ATTACCGCGGCTGCTGG-3′) and 340F (5′*-*CCCTAHGGGGYGCASCA-3) and 915R (5′-GWGCYCCCCCGYCAATTC-3′) targeting the regions V1-V3 and V3-V5 of bacterial and archaeal *16S rRNA* genes, respectively. PCR reactions were performed following the protocol described elsewhere^80^. Reactions were set up in 25 μL volumes containing 15 ng of DNA, 0.5 μM primers and 1X Phusion High-Fidelity PCR Master Mix (Thermo Fisher Scientific, Waltham, MA USA). PCR settings were as follows: initial denaturation at 98 °C for 10 s, 30 cycles of 98 °C for 1 s, 60 °C for 5 s, 72 °C for 15 s and final elongation at 72 °C for 1 min. The amplicon libraries were purified using the Agencourt^®^ AMpure XP bead protocol (Beckmann Coulter, USA). Sequencing libraries were prepared from the purified amplicon libraries using a second PCR. Each PCR reaction (50 μL) contained Phusion High-Fidelity PCR Master Mix (Thermo Fisher Scientific, Waltham, MA USA), Nextera XT Index Primers and 5 μL of amplicon library template. PCR settings: initial denaturation at 98 °C for 10 s, 8 cycles of 98 °C for 1 s, 55 °C for 5 s, 72 °C for 15 s and final elongation at 72 °C for 1 min. The amplicon libraries were purified using the Agencourt^®^ AMpure XP bead protocol (Beckmann Coulter, USA). Library concentration was measured with Qubit 3.0 Fluorometer (Thermo Fisher Scientific, Waltham, MA USA). Purified libraries were pooled in equimolar concentrations and diluted to 4 nM. Samples were paired end sequenced (2 × 301 bp) on a MiSeq platform (Illumina) using a MiSeq Reagent kit v3, 600 cycles (Illumina, USA) following standard guidelines for preparing and loading samples. 10% Phix control library was spiked in to overcome low complexity issue often observed with amplicon samples.

After checking read quality with fastqc, the sequences were processed and analyzed using QIIME2 v. 2018.2. The reads were demultiplexed using demux plugin (https://github.com/qiime2/q2-demux) and the primer sequences were removed by using cutadapt plugin (https://github.com/qiime2/q2-cutadapt). The demultiplexed reads were denoised, dereplicated and chimera-filtered using DADA2 algorithm. Additionally, DADA2 resolved amplicon sequence variants (ASVs), which infer the biological sequences in the samples prior to the introduction of amplification and sequencing errors and distinguish sequence variants differing by as little as one nucleotide^81^. The reads were subsampled and rarefied at the same number for each sample by using the feature-table rarefy plugin. Taxonomy was assigned to ASVs using a pre-trained naïve-Bayes classifier based on the 16S rRNA gene database at 99% similarity of the Silva132 release.

### Real-time quantification of *mcrA* genes

The quantification of functional genes involved in the methane production pathway (*mcrA* gene) was performed by qPCR using Sso Advanced Universal SYBR Green Supermix (BIO-RAD, United States) on a CFX96 Touch Real-time PCR detection system. The primer pair mlas (5′- GGTGGTGTMGGDTTCACMCARTA -3′) and mcrA-rev (5′- CGTTCATBGCGTAGTTVGGRTAGT -3′) was used for the detection of *mcrA* gene. Standard curves for the absolute quantification were constructed by using the long amplicons method. Melting curves were performed for each reaction to confirm the purity of amplified products^82^.

### Chlorophyll-a signals

Chlorophyll-a (Chl-a) was assessed after overnight cold 90% acetone–methanol (5:1, by volume) extraction^83^ of plankton retained on a Whatman GFC glass fiber filter after filtering 100 ml of a freshly collected water sample, stored not longer than 3 h, transported in a portable cool box. After boiling (2 min at 65 °C), the extracts were centrifuged and readings of the clear supernatant were obtained using a HACH DREL 2900 spectrophotometer set in wavelength scan mode (320–882 nm). The value retained corresponded to the highest peak recorded in the region 663–665 nm. Absorbance conversion to μg L^−1^ was carried out considering a specific absorption coefficient of 84.1 ml μg^−1^ cm^−1^.

### Epifluorescence and confocal microscopy

Total prokaryotic abundance was estimated by DAPI staining. Bacteria and Archaea abundances were determined by Catalyzed Reported Deposition—Fluorescence in situ Hybridization (CARD-FISH)^84^. Specific rRNA-target Horseradish peroxidase labeled oligonucleotidic probes (Biomers, Ulm, Germany) targeted Bacteria (EUB338 I-III), and Archaea (ARCH915). Stained filter sections were inspected on a Leica DM LB30 epifluorescence microscope (Leica Microsystems GmbH, Wetzlar, Germany) at ×1000 magnification. At least 300 cells were counted in >10 microscopic fields randomly selected across the filter sections. The relative abundance of hybridized cells was estimated as the ratio of hybridized cells to total DAPI-stained cells. Among the total EUB-positive cells, Cyanobacteria were discriminated by their red autofluorescence (excitation wavelength 550 nm). According to dominant cell morphologies, it was possible to distinguish between *Cyanobium*-like and *Synechocystis*-like cells. In order to visualize specific cells within the 3D structure of the aggregates, CARD-FISH was combined with confocal laser scanning microscopy (CSLM; Olympus FV1000). The hybridized Archaea cells were excited with the 488 nm line of an Ar laser (excitation) and observed in the green channel from 500 to 530 nm (emission). Cyanobacteria were excited with the 543-nm line of a He−Ne laser and observed in the red channel from 550 to 660 nm. The three-dimensional reconstruction of CSLM images was elaborated by IMARIS 7.6 (Bitplane, Switzerland).

### Flow cytometry

The abundance of microbial free-living cells and aggregates was assessed by an A50-micro flow cytometer, equipped with a 488-nm solid-state laser (Apogee Flow System, Hertfordshire, England). Absolute volumetric counts were performed by staining with SYBR Green I (1:10,000 dilution; Molecular Probes, Invitrogen). A threshold was set to the green channel and samples were run at low flow rate (<1000 events per s^−1^). Light scattering signals (i.e., forward and side scatter), and green fluorescence (530/30 nm) were registered for the characterization of each single cytometric event. Photomultiplier voltages and gating strategy were set using control water samples containing mainly single cells, and performed either by epifluorescence microscopy or flow cytometry^85^. Fixed gates were designed to discriminate between free-living cells and aggregates according to their signatures in a side scatter vs. green fluorescence plot^86^. Total microbial aggregates were back-gated on a forward scatter histogram plot and divided into submicrometric and micrometric particles, respectively showing forward scatter intensities lower and higher than those of 1-µm size calibration beads used as internal standard. Data visualization and extraction were computed with Apogee Histogram (v89.0—Apogee Flow System).

### Statistics and reproducibility

Non-parametric multivariate analysis of variance (one-way PERMANOVA) was used to test differences between water layers and sediments (OSW vs ASW vs BW vs Sed) in all major physical, chemical, and microbial parameters. Spearman’s rank correlation after inter sample rank ordination of SPE-DOM molecule was calculated to explore the relationship between DOM chemodiversity with the methane concentrations. PCoA, based on a Bray-Curtis similarity matrix, was applied to visualize all identified microbial taxa in an ordination plot, along with the percentage variance accounted for by the first two components. Data elaborations were computed using PAST (version 4.0)^87^.

### Reporting summary

Further information on research design is available in the Nature Research Reporting Summary linked to this article.

## Supplementary information

Supplementary Information

Reporting Summary

## Data Availability

Sequencing dataset is available through the Sequence Read Archive (SRA) under accession PRJNA731062. Flow cytometry.fcs files are available at the Flow Repository identifier: https://flowrepository.org/id/FR-FCM-Z3T7. All other data (geochemical variables, abundance of microbial cells and genes) are available from the corresponding author on reasonable request.
