# Supplementary material for: High concentrations of dissolved biogenic methane associated with cyanobacterial blooms in East African lake surface water
Source: Commun Biol. 2021 Jul 7;4:845. doi: 10.1038/s42003-021-02365-x (PMC8263762; doi:10.1038/s42003-021-02365-x)
Supplement: Supplementary file 1 — Supplementary Information [file 42003_2021_2365_MOESM1_ESM.pdf]

High concentrations of dissolved biogenic methane associated with cyanobacterial blooms in East African lake surface water

Stefano Fazi, Stefano Amalfitano, Stefania Venturi, Nic Pacini, Eusebi Vazquez, Lydia A. Olaka, Franco Tassi, Simona Crognale, Peter Herzsprung, Oliver J. Lechtenfeld, Jacopo Cabassi, Francesco Capecchiacci, Simona Rossetti, Mikhail Iakimov, Orlando Vaselli, David M. Harper, Andrea Butturini

SUPPLEMENTARY MATERIALS

Appendix 1

Water chemical stratification

In Supplementary Figure 1, the profile of water density was calculated from the composition of solutes following the method proposed by Boehrer et al. (2010) [1]. A sharp density gradient was recognized between the mixolimnion and the monimolimnion, restricting any vertical exchange between the two water layers and sustaining meromixis. This evidence was supported by the analysis of water stable isotopes.

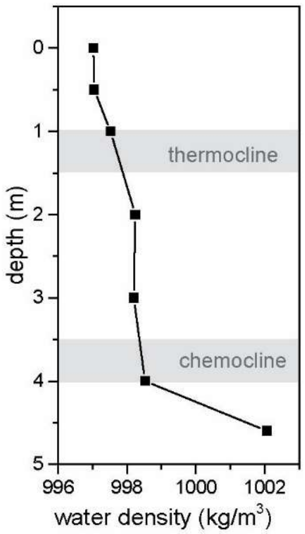

Supplementary Figure 1 Vertical profile of water density, calculated according to Boehrer et al. (2010) [1].

As shown in Supplementary Figure 2, the measured δD-H_2O and $\delta^{18}O$-H_2O values of Sonachi waters were significantly higher than those (i) measured in local meteoric waters [2,3], and (ii) derived for rainfall at the lake and the crater rim elevation according to the altitude effect relationships suggested by Allen et al. [2], i.e. about -18 and -3.6 ‰ vs. V-SMOW. Moreover, although hydrologically connected to Lake Naivasha via groundwater seepage [4], waters from Sonachi were notably enriched in heavy isotopes with respect to Naivasha (δD-H_2O and $\delta^{18}O$-H_2O around +36.5 and +5.6 ‰ vs. V-SMOW, respectively; own unpublished data). In Fig. A2, Sonachi waters were relatively well aligned with Naivasha and local meteoric waters. The alignment had a slope of ~5, in agreement with that recognized by Odada [5] for East Africa lake waters, and roughly corresponded to the Kenya Rift Valley Meteoric Line (KRVML: $\partial D = 5.56 \times \partial^{18}O + 2.04$) and the Kenya Rift Valley Groundwater Line (KRVGL: $\partial D = 5.49 \times \partial^{18}O + 0.08$) [2]. Such a trajectory clearly indicated that, despite having a meteoric origin, Sonachi waters were largely affected by evaporation processes with respect to both direct rainfall and inflowing waters from the Naivasha basin. During evaporation, a vapor depleted in the light ^{16}O and ^{1}H isotopes is produced, whereas the residual water becomes progressively enriched in the heavier ^{18}O and D. The sharp isotopic shift towards heavier δD-H_2O and $\delta^{18}O$-H_2O values observed between the monimolimnion and the mixolimnion suggested that a high evaporation rate affected the shallowest water layers and that no vertical mixing occurred between the monimolimnion and mixolimnion waters. Lack of vertical turnover and the resultant stratification of the water column into the shallow mixed layer and the permanently separated monimolimnion produced strong differences in water chemistry.

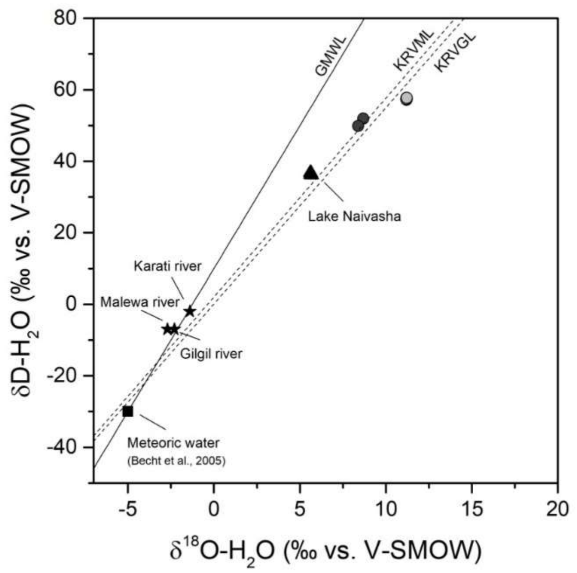

Supplementary Figure 2 δD-H_2O vs. $\delta^{18}O$-H_2O binary diagram for monimolimnetic (dark grey circles) and mixolimnetic (light grey circles) waters from Lake Sonachi. The global meteoric water line (GMWL; [6]) is shown, together with the Kenya Rift Valley Meteoric Line and the Kenya Rift Valley Groundwater Line (KRVML and KRVGL, respectively) [2]. The isotopic composition of waters from Lake Naivasha (own unpublished data), its tributary rivers (Karati, Malewa, Gilgil [7]), and local meteoric waters [3] are also reported for comparison.

Supplementary Table 1 Methane concentration in surface oxic waters (descending order) of lakes distributed worldwide, across a range of climatic regions (i.e., arctic, boreal, Mediterranean, temperate, tropical), geochemical setting, morphology (e.g., area, depth), and trophic status (e.g., Chl-a, DOC). All data are obtained from the cited literature studies. Data are ordered according to descending CH_4 concentration.

Climatic region	Geochemistry	Lake type	CH_4 (nM)	Area (km^2)	Depth (m)	Chl-a (mg/L)	DOC (ppm)	Reference
Tropical	Alkaline	Meromictic	151000	0.18	4.6	88.0	105.9	This study
Boreal	Freshwater	Lake	36875	0.023	6	1.5.	n.a.	Sepulveda-Jauregui et al. 2015 [8]
Temperate	Freshwater	Meromictic	30000	1.26	25	n.a.	n.a.	Roland et al. 2017 [9]
Temperate	Freshwater	Lake	17600	0.07	n.a.	n.a.	n.a.	Rinta et al. 2015 [10]
Boreal	Freshwater	Small lake	11000	0.0035	3	11.2	22.3	Taipale et al. 2011 [11]
Temperate	Freshwater	Meromictic	10000	0.05	14	45.0	n.a.	Lambrecht et al. 2020 [12]
Temperate	Freshwater	Lake	8695	0.55	2	n.a.	n.a.	Fernández et al. 2016 [13]
Temperate	Freshwater	Lake	8592	0.55	2	n.a.	n.a.	Fernández et al. 2016 [13]
Temperate	Freshwater	Lake	8000	0.25	n.a.	n.a.	n.a.	Schilder et al. 2016 [14]
Mediterranean	Freshwater	Meromictic	7800	0.16	35	n.a.	n.a.	Cabassi et al. 2013 [15]
Temperate	Freshwater	Lake	5673	16.92	43	n.a.	n.a.	Fernández et al. 2016 [13]
Boreal	Freshwater	Lake	5625	0.025	4.8	45.9	n.a.	Sepulveda-Jauregui et al. 2015 [8]
Temperate	Freshwater	Lake	5287	13.44	21	n.a.	n.a.	Fernández et al. 2016 [13]
Mediterranean	Freshwater	Meromictic	5100	0.41	38	n.a.	n.a.	Cabassi et al. 2013 [15]
Boreal	Freshwater	Lake	5000	0.575	11.5	7.2	52.2	Sepulveda-Jauregui et al. 2015 [8]
Temperate	Freshwater	Lake	5000	1.4	n.a.	n.a.	n.a.	Bédard & Knowles 1997 [16]
Temperate	Freshwater	Lake	5000	4	12	n.a.	5.3	West et al. 2012 [17]
Temperate	Freshwater	Lake	4601	0.55	2	n.a.	n.a.	Fernández et al. 2016 [13]
Temperate	Freshwater	Lake	4426	31.46	43	n.a.	n.a.	Fernández et al. 2016 [13]
Temperate	Freshwater	Lake	4424	13.44	21	n.a.	n.a.	Fernández et al. 2016 [13]
Boreal	Freshwater	Lake	4375	0.051	7	3.4	0.71	Sepulveda-Jauregui et al. 2015 [8]
Boreal	Freshwater	Lake	3900	0.003	n.a.	n.a.	n.a.	Rinta et al. 2015 [10]
Temperate	Freshwater	Lake	3600	0.09	n.a.	n.a.	n.a.	Rinta et al. 2015 [10]
Temperate	Freshwater	Small lake	3500	0.0416	32	n.a.	2.5	Hamilton et al. 1994 [18]
Temperate	Freshwater	Lake	3500	0.655	5.8	n.a.	4.5	Striegl & Michmerhuizen 1998 [19]
Boreal	Freshwater	Lake	3300	0.06	n.a.	n.a.	n.a.	Rinta et al. 2015 [10]
Temperate	Freshwater	Lake	3300	0.24	n.a.	n.a.	n.a.	Rinta et al. 2015 [10]
Boreal	Freshwater	Shallow lake	3125	0.35	2.2	2.6	16.48	Martinez-Cruz et al. 2015 [20]
Tropical	Freshwater	Lake	3000	13	14	n.a.	2.4	Kankaala et al. 2013 [21]

Artic	Freshwater	Lake	2800	0.215	19	n.a.	24.9	Northington & Saros 2016 [22]
Temperate	Freshwater	Lake	2800	0.05	n.a.	n.a.	n.a.	Rinta et al. 2015 [10]
Temperate	Freshwater	Lake	2650	0.55	2	n.a.	n.a.	Fernández et al. 2016 [13]
Boreal	Freshwater	Small lake	2600	0.004	4	n.a.	25	Kankaala et al. 2013 [21]
Boreal	Freshwater	Lake	2500	0.026	6.4	1.3	0.18	Sepulveda-Jauregui et al. 2015 [8]
Boreal	Freshwater	Lake	2500	0.384	6.3	10.9	2.58	Sepulveda-Jauregui et al. 2015 [8]
Temperate	Freshwater	Lake	2340	16.92	24	n.a.	n.a.	Fernández et al. 2016 [13]
Temperate	Freshwater	Lake	2176	0.16	10	8.5	n.a.	Fernández et al. 2016 [13]
Temperate	Freshwater	Lake	2118	0.55	2	n.a.	n.a.	Fernández et al. 2016 [13]
Boreal	Freshwater	Small lake	2100	0.008	6.5	n.a.	10	Kankaala et al. 2013 [21]
Boreal	Freshwater	Lake	2100	0.009	n.a.	n.a.	n.a.	Rinta et al. 2015 [10]
Temperate	Freshwater	Lake	2068	0.55	2	n.a.	n.a.	Fernández et al. 2016 [13]
Temperate	Freshwater	Lake	2001	0.55	2	n.a.	n.a.	Fernández et al. 2016 [13]
Boreal	Freshwater	Shallow lake	2000	0.23	1.5	n.a.	10	Repo et al. 2007 [23]
Temperate	Freshwater	Small lake	2000	0.017	12	4.3	3.65	Bastviken et al. 2008 [24]
Boreal	Freshwater	Lake	1875	1.45	24.1	1.5	0.7	Sepulveda-Jauregui et al. 2015 [8]
Boreal	Freshwater	Lake	1875	0.077	9	54.0	n.a.	Sepulveda-Jauregui et al. 2015 [8]
Boreal	Freshwater	Lake	1875	0.63	5.5	12.6	1.05	Sepulveda-Jauregui et al. 2015 [8]
Boreal	Freshwater	Lake	1875	1.074	30	3.7	n.a.	Sepulveda-Jauregui et al. 2015 [8]
Boreal	Freshwater	Lake	1875	0.471	28	9.9	n.a.	Sepulveda-Jauregui et al. 2015 [8]
Temperate	Freshwater	Lake	1800	0.45	n.a.	n.a.	n.a.	Rinta et al. 2015 [10]
Temperate	Freshwater	Lake	1718	0.64	17	10.2	n.a.	Fernández et al. 2016 [13]
Boreal	Freshwater	Small lake	1500	0.0035	3	7.2	26.8	Taipale et al. 2011 [11]
Temperate	Freshwater	Lake	1500	0.01	3.8	300.0	n.a.	Casper et al. 2000 [25]
Temperate	Freshwater	Lake	1500	536	n.a.	n.a.	n.a.	Hofmann et al. 2010 [26]
Temperate	Freshwater	Lake	1500	0.23	26	n.a.	5.2	Vachon et al. 2020 [27]
Temperate	Freshwater	Lake	1414	13.44	21	n.a.	n.a.	Fernández et al. 2016 [13]
Temperate	Freshwater	Lake	1400	4.6	n.a.	n.a.	n.a.	Grossart et al. 2011 [28]
Temperate	Freshwater	Lake	1400	0.19	n.a.	n.a.	n.a.	Rinta et al. 2015 [10]
Temperate	Freshwater	Lake	1400	0.25	n.a.	n.a.	n.a.	Rinta et al. 2015 [10]
Temperate	Freshwater	Small lake	1350	0.026	20	3.6	4.51	Bastviken et al. 2008 [24]
Temperate	Freshwater	Lake	1294	31.46	43	n.a.	n.a.	Fernández et al. 2016 [13]
Boreal	Freshwater	Lake	1250	0.21	24.2	7.4	2.38	Sepulveda-Jauregui et al. 2015 [8]
Temperate	Freshwater	Lake	1204	1.05	13.5	8.8	n.a.	Fernández et al. 2016 [13]
Temperate	Freshwater	Lake	1200	0.2	n.a.	n.a.	n.a.	Rinta et al. 2015 [10]
Temperate	Freshwater	Lake	1175	16.92	24	n.a.	n.a.	Fernández et al. 2016 [13]
Temperate	Freshwater	Lake	1106	0.55	2	n.a.	n.a.	Fernández et al. 2016 [13]
Boreal	Freshwater	Small lake	1100	0.004	11.5	n.a.	22	Kankaala et al. 2013 [21]

Boreal	Freshwater	Lake	1100	23.7	9	n.a.	10.7	Denfeld et al. 2016 [29]
Temperate	Freshwater	Lake	1100	0.34	n.a.	n.a.	n.a.	Rinta et al. 2015 [10]
Boreal	Freshwater	Small lake	1000	0.017	4.3	1.5	6.6	Thottathil et al. 2019 [30]
Temperate	Freshwater	Lake	1000	0.75	n.a.	n.a.	n.a.	Rinta et al. 2015 [10]
Temperate	Freshwater	Lake	1000	0.14	n.a.	n.a.	n.a.	Rinta et al. 2015 [10]
Temperate	Freshwater	Lake	1000	0.04	n.a.	n.a.	n.a.	Rinta et al. 2015 [10]
Temperate	Freshwater	Lake	1000	0.14	n.a.	n.a.	n.a.	Rinta et al. 2015 [10]
Temperate	Freshwater	Meromictic	1000	1.26	25	n.a.	n.a.	Roland et al. 2017 [9]
Boreal	Saline	Meromictic	1000	0.36	18	n.a.	n.a.	Savvichev et al. 2020 [31]
Temperate	Freshwater	Meromictic	1000	0.26	20	n.a.	n.a.	Milucka et al. 2015 [32]
Tropical	Freshwater	Lake	1000	70	7	n.a.	n.a.	Barbosa et al. 2018 [33]
Boreal	Freshwater	Lake	970	0.24	3.4	n.a.	15.4	Denfeld et al. 2016 [29]
Boreal	Freshwater	Small lake	900	0.012	11	n.a.	19	Kankaala et al. 2013 [21]
Temperate	Freshwater	Lake	850	0.64	17	10.2	n.a.	Fernández et al. 2016 [13]
Boreal	Freshwater	Small lake	833	0.07	6.5	n.a.	10	Huotari 2011 [34]
Boreal	Freshwater	Shallow lake	800	0.722	1.2	n.a.	16.2	N.a.tchimuthu et al. 2016 [35]
Artic	Freshwater	Lake	800	0.246	11	n.a.	42.8	Northington & Saros 2016 [22]
Boreal	Freshwater	Lake	800	0.44	8.3	1.5	4.9	Thottathil et al. 2019 [30]
Temperate	Freshwater	Lake	800	10.3	n.a.	n.a.	n.a.	Donis et al. 2017 [36]
Temperate	Freshwater	Lake	800	0.1	10	n.a.	n.a.	DelSontro et al. 2018 [37]
Temperate	Freshwater	Small lake	760	0.014	12	4.3	5.1	West et al. 2016 [38]
Boreal	Freshwater	Small lake	700	0.008	8	n.a.	11	Kankaala et al. 2013 [21]
Artic	Freshwater	Small lake	700	0.146	10	n.a.	50.4	Northington & Saros 2016 [22]
Boreal	Freshwater	Small lake	700	0.01	9.8	2.0	7.7	Thottathil et al. 2019 [30]
Temperate	Alkaline	Lake	700	185	17	n.a.	n.a.	Miller & Oremland 1988 [39]
Temperate	Freshwater	Lake	700	0.44	n.a.	n.a.	n.a.	Rinta et al. 2015 [10]
Temperate	Freshwater	Lake	674	0.16	10	8.5	n.a.	Fernández et al. 2016 [13]
Temperate	Freshwater	Lake	653	0.19	20	n.a.	n.a.	DelSontro et al. 2018 [37]
Boreal	Freshwater	Shallow lake	650	6.5	0.8	n.a.	19.7	Pokrovsky et al. 2013 [40]
Boreal	Freshwater	Small lake	610	0.16	2.6	n.a.	14.3	Denfeld et al. 2016 [29]
Artic	Freshwater	Small lake	600	0.033	13	n.a.	9.3	Northington & Saros 2016 [22]
Artic	Freshwater	Small lake	600	0.07	14	n.a.	18.8	Northington & Saros 2016 [22]
Temperate	Freshwater	Lake	600	0.02	n.a.	n.a.	n.a.	Rinta et al. 2015 [10]
Temperate	Freshwater	Lake	565	0.16	10	8.5	n.a.	Fernández et al. 2016 [13]
Temperate	Freshwater	Lake	560	0.697	12.2	4.4	6.5	West et al. 2016 [38]
Temperate	Freshwater	Lake	540	64.7	175	n.a.	n.a.	Fernández et al. 2016 [13]
Temperate	Freshwater	Lake	508	0.26	20	n.a.	n.a.	DelSontro et al. 2018 [37]
Tropical	Freshwater	Lake	500	350	19	n.a.	1.8	Kankaala et al. 2013 [21]

Artic	Freshwater	Lake	500	0.243	18	n.a.	19.5	Northington & Saros 2016 [22]
Boreal	Freshwater	Lake	500	0.05	n.a.	n.a.	n.a.	Rinta et al. 2015 [10]
Boreal	Freshwater	Lake	500	0.004	n.a.	n.a.	n.a.	Rinta et al. 2015 [10]
Temperate	Freshwater	Meromictic	500	0.01	23	3.0	n.a.	Lambrecht et al. 2020 [12]
Boreal	Freshwater	Shallow lake	440	0.24	1.3	n.a.	27.1	Denfeld et al. 2016 [29]
Boreal	Freshwater	Lake	430	2.46	6.1	n.a.	6.8	Denfeld et al. 2016 [29]
Temperate	Freshwater	Lake	424	0.16	10	8.5	n.a.	Fernández et al. 2016 [13]
Boreal	Freshwater	Small lake	400	0.042	6.5	n.a.	12	Kankaala et al. 2013 [21]
Boreal	Freshwater	Small lake	400	0.064	5	n.a.	14	Kankaala et al. 2013 [21]
Boreal	Freshwater	Small lake	400	0.041	6.5	21.7	11	Huotari 2011 [34]
Boreal	Freshwater	Lake	400	0.04	n.a.	n.a.	n.a.	Rinta et al. 2015 [10]
Boreal	Freshwater	Lake	400	0.26	n.a.	n.a.	n.a.	Rinta et al. 2015 [10]
Boreal	Freshwater	Lake	400	0.16	n.a.	n.a.	n.a.	Rinta et al. 2015 [10]
Temperate	Freshwater	Lake	400	46	81	n.a.	n.a.	Fernández et al. 2016 [13]
Tropical	Freshwater	Shallow lake	400	760	2.5	15.0	6.5	Zhang et al. 2021 [41]
Temperate	Freshwater	Lake	372	0.64	17	10.2	n.a.	Fernández et al. 2016 [13]
Boreal	Freshwater	Small lake	350	0.062	5	n.a.	22.5	N.a.tchimuthu et al. 2016 [35]
Temperate	Freshwater	Lake	350	6.9	n.a.	n.a.	n.a.	Vagle et al. 2010 [42]
Temperate	Freshwater	Lake	347	0.46	16	13.1	n.a.	Khatun et al. 2019 [43]
Boreal	Freshwater	Small lake	335	0.0001	3.2	6.0	8.9	Huttunen et al. 2003 [44]
Temperate	Freshwater	Small lake	320	0.002	8	32.3	23.4	West et al. 2016 [38]
Boreal	Freshwater	Small lake	312	0.1	7	n.a.	3.9	Shirokova et al. 2013 [45]
Temperate	Freshwater	Lake	308	1.4	30	7.0	n.a.	Khatun et al. 2019 [43]
Temperate	Freshwater	Lake	300	2.1	73	2.2	0.922	Khatun et al. 2020 [46]
Boreal	Freshwater	Lake	300	0.256	20.3	1.1	2.7	Thottathil et al. 2019 [30]
Boreal	Freshwater	Small lake	300	0.179	11.4	0.9	4.9	Thottathil et al. 2019 [30]
Boreal	Freshwater	Small lake	300	0.008	8.2	2.9	11	Thottathil et al. 2019 [30]
Temperate	Freshwater	Lake	300	220	n.a.	n.a.	n.a.	Utsumi et al. 1998 [47]
Boreal	Freshwater	Lake	300	0.033	n.a.	n.a.	n.a.	Rinta et al. 2015 [10]
Boreal	Freshwater	Lake	300	0.99	n.a.	n.a.	n.a.	Rinta et al. 2015 [10]
Temperate	Freshwater	Lake	281	0.16	10	8.5	n.a.	Fernández et al. 2016 [13]
Temperate	Freshwater	Lake	280	0.259	18.5	4.0	4.5	West et al. 2016 [38]
Temperate	Freshwater	Lake	280	0.07	11	n.a.	n.a.	DelSontro et al. 2018 [37]
Temperate	Freshwater	Lake	280	0.044	1.8	6.3	11.1	West et al. 2016 [38]
Temperate	Freshwater	Lake	263	0.07	9.5	3.2	n.a.	Khatun et al. 2019 [43]
Temperate	Freshwater	Lake	261	0.43	64	1.3	n.a.	Khatun et al. 2019 [43]
Temperate	Freshwater	Lake	254	0.02	11	15.2	n.a.	Khatun et al. 2019 [43]
Temperate	Freshwater	Small lake	250	0.011	3.5	20.7	19.5	West et al. 2016 [38]

Temperate	Freshwater	Small lake	250	0.016	15	7.0	13.4	West et al. 2016 [38]
Tropical	Freshwater	Lake	250	32900	1400	n.a.	n.a.	Rudd 1980 [48]
Temperate	Freshwater	Lake	250	0.016	15	7.0	13.4	West et al. 2016 [38]
Temperate	Freshwater	Small lake	240	0.162	12	12.8	9.4	West et al. 2016 [38]
Temperate	Freshwater	Small lake	240	0.012	7.9	16.7	18.2	West et al. 2016 [38]
Temperate	Freshwater	Small lake	240	0.049	14	8.1	7.4	West et al. 2016 [38]
Temperate	Freshwater	Lake	240	0.012	7.9	16.7	18.2	West et al. 2016 [38]
Temperate	Freshwater	Lake	235	3.5	17	n.a.	n.a.	DelSontro et al. 2018 [37]
Temperate	Freshwater	Lake	231	5.7	16	4.1	n.a.	Khatun et al. 2019 [43]
Boreal	Freshwater	Shallow lake	230	4	0.85	n.a.	33.3	Pokrovsky et al 2013 [40]
Temperate	Alkaline	Lake	230	3.5	21	n.a.	n.a.	Miller & Oremland 1988 [39]
Temperate	Freshwater	Lake	202	0.07	7	12.3	n.a.	Khatun et al. 2019 [43]
Temperate	Freshwater	Lake	200	7	44	1.7	1.007	Khatun et al. 2020 [46]
Temperate	Freshwater	Lake	200	4.5	39	1.8	1.123	Khatun et al. 2020 [46]
Temperate	Freshwater	Lake	200	10.8	31	3.8	0.978	Khatun et al. 2020 [46]
Temperate	Freshwater	Lake	200	0.367	20	5.1	1.86	Riera et al. 1999 [49]
Temperate	Freshwater	Lake	200	0.64	20	6.7	3.24	Riera et al. 1999 [49]
Temperate	Freshwater	Small lake	200	0.005	2.5	28.0	9.288	Riera et al. 1999 [49]
Temperate	Freshwater	Small lake	200	0.011	7.9	21.0	20.74	Riera et al. 1999 [49]
Temperate	Freshwater	Small lake	200	0.026	18	3.4	6.4	West et al. 2016 [38]
Artic	Freshwater	Small lake	200	0.085	18	n.a.	5.3	Northington & Saros 2016 [22]
Artic	Freshwater	Small lake	200	0.092	11	n.a.	21.6	Northington & Saros 2016 [22]
Artic	Freshwater	Lake	200	0.368	12	n.a.	26.1	Northington & Saros 2016 [22]
Temperate	Freshwater	Lake	200	0.367	20	5.1	1.86	Riera et al. 1999 [49]
Temperate	Freshwater	Lake	200	0.64	20	6.7	3.24	Riera et al. 1999 [49]
Boreal	Freshwater	Lake	200	0.03	n.a.	n.a.	n.a.	Rinta et al. 2015 [10]
Boreal	Freshwater	Lake	200	0.23	n.a.	n.a.	n.a.	Rinta et al. 2015 [10]
Boreal	Freshwater	Lake	200	0.62	n.a.	n.a.	n.a.	Rinta et al. 2015 [10]
Boreal	Freshwater	Lake	200	0.96	n.a.	n.a.	n.a.	Rinta et al. 2015 [10]
Boreal	Freshwater	Lake	200	0.07	n.a.	n.a.	n.a.	Rinta et al. 2015 [10]
Temperate	Freshwater	Lake	200	1.14	n.a.	n.a.	n.a.	Rinta et al. 2015 [10]
Temperate	Freshwater	Small lake	190	0.042	6.1	2.3	8.6	West et al. 2016 [38]
Temperate	Freshwater	Lake	190	0.042	6.1	2.3	8.6	West et al. 2016 [38]
Temperate	Freshwater	Lake	182	1.9	9	n.a.	n.a.	DelSontro et al. 2018 [37]
Temperate	Freshwater	Small lake	180	0.059	6.7	7.2	22.6	West et al. 2016 [38]
Temperate	Freshwater	Lake	170	0.296	4.9	40.2	6.6	West et al. 2016 [38]
Temperate	Freshwater	Small lake	170	0.008	7.6	13.8	23	West et al. 2016 [38]
Temperate	Freshwater	Lake	168	4.46	39	2.6	n.a.	Khatun et al. 2019 [43]

Temperate	Alkaline	Lake	150	1.5	63	n.a.	n.a.	Miller & Oremland 1988 [39]
Temperate	Freshwater	Lake	149	6.99	44	4.4	n.a.	Khatun et al. 2019 [43]
Boreal	Freshwater	Shallow lake	143	0.35	0.8	n.a.	84	Shirokova et al. 2013 [45]
Temperate	Freshwater	Lake	131	5.3	27	n.a.	n.a.	DelSontro et al. 2018 [37]
Temperate	Freshwater	Small lake	130	0.057	13.7	8.0	14	West et al. 2016 [38]
Temperate	Freshwater	Lake	123	0.64	17	10.2	n.a.	Fernández et al. 2016 [13]
Temperate	Freshwater	Lake	123	0.12	7.7	13.0	n.a.	Khatun et al. 2019 [43]
Boreal	Freshwater	Lake	120	0.74	7	n.a.	14	Kankaala et al. 2013 [21]
Boreal	Freshwater	Lake	120	26	33	7.8	6.5	Linnaluoma 2012 [50]
Temperate	Freshwater	Lake	112	0.16	10	8.5	n.a.	Fernández et al. 2016 [13]
Temperate	Freshwater	Lake	107	0.07	8.6	2.0	n.a.	Khatun et al. 2019 [43]
Artic	Freshwater	Small lake	100	0.106	15	n.a.	7.9	Northington & Saros 2016 [22]
Artic	Freshwater	Lake	100	0.354	29	n.a.	8	Northington & Saros 2016 [22]
Artic	Freshwater	Lake	100	0.358	28	n.a.	19	Northington & Saros 2016 [22]
Boreal	Freshwater	Lake	100	1.67	n.a.	n.a.	n.a.	Rinta et al. 2015 [10]
Temperate	Freshwater	Meromictic	100	0.4	90	n.a.	n.a.	Lopes et al. 2011 [51]
Boreal	Freshwater	Lake	90	0.5	10	n.a.	10	Kankaala et al. 2013 [21]
Temperate	Freshwater	Lake	90	1.7	58	1.1	0.51	Khatun et al. 2020 [46]
Mediterranean	Freshwater	Meromictic	90	6	167	n.a.	n.a.	Cabassi et al. 2013 [15]
Temperate	Freshwater	Lake	89	1269	122	n.a.	n.a.	DelSontro et al. 2018 [37]
Boreal	Freshwater	Lake	70	6.53	30	8.1	7.6	Linn.a.luoma 2012 [50]
Temperate	Freshwater	Lake	67	22	36	n.a.	n.a.	DelSontro et al. 2018 [37]
Tropical	Freshwater	Lake	66	2370	n.a.	2.2	2.5	Borges et al. 2011 [52]
Boreal	Freshwater	Lake	61	6.53	30	n.a.	8	Kankaala et al. 2013 [21]
Temperate	Freshwater	Lake	59	2.13	73	2.7	n.a.	Khatun et al. 2019 [43]
Boreal	Freshwater	Shallow lake	50	15	0.8	n.a.	25.7	Pokrovsky et al. 2013 [40]
Temperate	Freshwater	Small lake	50	0.008	8	17.7	22.01	Bastviken et al. 2008 [24]
Temperate	Freshwater	Lake	50	48.7	280	n.a.	n.a.	Blees et al. 2015 [53]
Mediterranean	Freshwater	Meromictic	50	0.55	33	n.a.	n.a.	Tassi et al. 2018 [54]
Tropical	Alkaline	Shallow lake	44	1033	1.5	4.0	5.6	Ray 2013 [55]
Tropical	Freshwater	Shallow lake	44	1033	1.5	4.0	5.6	Ray 2013 [55]
Boreal	Freshwater	Shallow lake	40	15.9	0.9	n.a.	19.6	Pokrovsky et al. 2013 [40]
Boreal	Freshwater	Lake	40	0.58	6	n.a.	9.7	Denfeld et al. 2016 [29]
Boreal	Freshwater	Small lake	40	0.02	2.9	n.a.	28	Denfeld et al. 2016 [29]
Temperate	Freshwater	Lake	40	4.7	122	1.2	1.174	Khatun et al. 2020 [46]
Temperate	Freshwater	Lake	40	11.6	163	5.8	0.614	Khatun et al. 2020 [46]
Boreal	Freshwater	Lake	40	13.4	87	4.4	12.3	Linn.a.luoma 2012 [50]
Boreal	Freshwater	Shallow lake	40	12	1.7	16.0	n.a.	Larmola et al. 2004 [56]

Temperate	Freshwater	Lake	40	170	35	n.a.	n.a.	DelSontro et al. 2018 [37]
Temperate	Freshwater	Lake	36	17	70	n.a.	n.a.	DelSontro et al. 2018 [37]
Boreal	Freshwater	Lake	33	13.4	87	n.a.	13	Kankaala et al. 2013 [21]
Temperate	Freshwater	Lake	32	19009	220	n.a.	n.a.	DelSontro et al. 2018 [37]
Mediterranean	Freshwater	Meromictic	30	0.55	33	n.a.	n.a.	Cabassi et al. 2013 [15]
Temperate	Freshwater	Lake	28	4.65	122	1.0	n.a.	Khatun et al. 2019 [43]
Temperate	Freshwater	Lake	27	1.73	58	1.8	n.a.	Khatun et al. 2019 [43]
Temperate	Freshwater	Lake	25	26	30	n.a.	n.a.	DelSontro et al. 2018 [37]
Boreal	Freshwater	Lake	24	13.4	87	1.1	13.3	López Bellido et al. 2009 [57]
Temperate	Freshwater	Lake	24	13.4	87	1.1	13.3	Casper et al. 2000 [25]
Temperate	Freshwater	Lake	20	70.4	180	0.7	0.515	Khatun et al. 2020 [46]
Boreal	Freshwater	Lake	16	13.4	87	6.0	12.9	López Bellido et al. 2009 [57]
Temperate	Freshwater	Lake	16	13.4	87	6.0	12.9	Casper et al. 2000 [25]
Temperate	Freshwater	Lake	15	78.8	363	0.5	0.320	Khatun et al. 2020 [46]
Boreal	Freshwater	Lake	14	35	32	n.a.	6	Kankaala et al. 2013 [21]
Temperate	Freshwater	Lake	9	1065	65	n.a.	n.a.	DelSontro et al. 2018 [37]
Boreal	Freshwater	Lake	5	31700	1600	1.3	1.2	Schmid et al. 2007 [58]

n.a. = not available

Supplementary Table 2 Chemical composition of the main solutes, reduced sulfur species (as ΣS^{2-} in mg L^{-1}) and trace elements in water samples from Lake Sonachi. δD-H_2O, $\delta^{18}O$-H_2O, and $\delta^{13}C$-TDIC values are also reported.

**Depth (m)**	**0**	**0.5**	**1**	**2**	**3**	**4**	**4.5**
HCO_3^- (mg L^{-1})	4033	4280	4005	4057	3909	6764	9523
CO_3^{2-} (mg L^{-1})	1044	1159	1036	1125	1135	1456	2196
F^- (mg L^{-1})	105	120	130	109	135	143	183
Cl^- (mg L^{-1})	268	222	298	322	249	294	472
Br^- (mg L^{-1})	2.8	2.2	3.0	3.2	2.5	3.0	4.4
SO_4^{2-} (mg L^{-1})	99.6	89.2	106	112	95.3	123	141
Ca^{2+} (mg L^{-1})	3.3	4.6	3.5	3.1	2.9	4.6	b.d.l.
Mg^{2+} (mg L^{-1})	2.0	3.3	2.3	2.1	2.0	1.2	b.d.l.
Na^+ (mg L^{-1})	2423	2489	2496	2509	2485	3359	5276
K^+ (mg L^{-1})	309	326	327	336	322	415	640
Si (mg L^{-1})	50.7	54.3	53.9	54.7	54.1	64.4	77.7
ΣS^{2-} (mg L^{-1})	7.7	7.8	7.9	8.1	8.2	37.9	62.9
Mn (μg L^{-1})	4.7	3.9	5.3	3.8	4.4	3.2	3.3
Fe (μg L^{-1})	16.2	9.3	29.5	13.5	37.5	19.0	30.2
Co (μg L^{-1})	1.1	0.9	0.9	1.1	1.2	1.6	2.5
Ni (μg L^{-1})	0.9	0.4	0.3	0.4	0.5	3.0	5.8
Cu (μg L^{-1})	4.7	3.6	3.6	3.4	4.1	4.4	3.3
Zn (μg L^{-1})	13.1	9.4	7.8	8.2	7.9	4.8	3.0
Pb (μg L^{-1})	b.d.l.	b.d.l.	b.d.l.	b.d.l.	b.d.l.	3.2	b.d.l.
Cd (μg L^{-1})	1.1	0.7	0.6	0.7	0.7	0.7	0.6
Sr (μg L^{-1})	64.0	63.9	62.9	61.7	64.5	54.9	60.0
Li (μg L^{-1})	176	173	161	154	163	128	159
Ba (μg L^{-1})	297	292	280	298	309	325	488
As (μg L^{-1})	44.9	44.9	45.0	46.3	46.5	62.4	95.0
Sb (μg L^{-1})	12.2	18.8	17.5	18.0	28.3	30.1	29.5
P-PO_4 (μg L^{-1})	50.6	45.1	46.4	54.7	80.0	3600	11360
N-NH_4+NH_3 (μg L^{-1})	35.1	28.02	32.93	35.1	39.46	b.l.d.	4.6
N-NO_2 (μg L^{-1})	0.6	1.74	2.32	1.62	3.01	1.39	5.09
N-NO_3 (μg L^{-1})	0.61	1.36	0.6	b.l.d.	0.7	3.05	b.l.d.
δD-H_2O (‰ vs. V-SMOW)	57.3	57.7	57.2	57.4	57.8	51.9	49.9
$\delta^{18}O$-H_2O (‰ vs. V-SMOW)	11.2	11.2	11.2	11.2	11.2	8.69	8.39
$\delta^{13}C$-TDIC (‰ vs. V-PDB)	2.85	3.01	3.06	3.00	2.91	7.73	10.7

Supplementary Table 3 DOM description. Average (±1SDE) values of bulk DOC and DON concentrations. Solid phase extraction (SPE) DOM molecular characterization according FT-ICT-MS analysis. CRAM = Carboxyl-rich alicyclic like molecules; CA = Condensed ad Aromatic like compounds; NOCS describes the nominal carbon oxidation state. Details in Butturini et al. [59].

Depth (m)	DOC (mM)	DON (mM)	CRAM		Aliphatic		Aromatic		CA	NOCS
			O poor	O rich	O poor	O rich	O poor	O rich		
0	7.3±0.56	0.75±2 10^{-3}	41.85	18.15	10.05	2.21	2.47	1.52	0.9	-0.34041
0.5	8.8±0.28	0.99±2 10^{-3}	39.54	19.36	9.25	2.11	2.42	1.72	0.85	-0.31592
1	8.4±0.12	0.88±2 10^{-3}	40.4	18.8	9.55	2.18	2.35	1.67	0.88	-0.32256
2	7.9±0.14	0.78±2 10^{-3}	40.02	19.05	9.5	2.19	2.38	1.68	0.87	-0.31842
3	8.0±0.11	0.79±2 10^{-3}	40.02	18.79	9.47	2.38	2.36	1.59	0.87	-0.32106
4	9.9±0.10	4.64± 10^{-4}	49.13	9.79	16.81	2.82	1.23	0.23	0.16	-0.50409
4.5	49.6±0.16	6.3±4 10^{-4}	39.59	16.15	12.6	2.94	1.59	0.63	0.29	-0.38641

Supplementary Table 4 Chemical composition of the main dissolved gases.

Depth (m)	0.5	1	2	3	4	4.5
CO_2 (µmol L^{-1})	1.1	2.4	13	96	126	115
N_2 (µmol L^{-1})	465	468	441	437	455	461
Ar (µmol L^{-1})	12	11	11	11	11	11
CH_4 (µmol L^{-1})	151	156	201	496	555	615
O_2 (µmol L^{-1})	47	13	7.2	5.6	4.7	3.8
H_2 (µmol L^{-1})	b.d.l.	b.d.l.	b.d.l.	0.5	0.5	0.8
He (µmol L^{-1})	0.002	0.002	0.002	0.003	0.002	0.003
P_{CO2} (atm)	0.00003	0.00006	0.00033	0.0024	0.0032	0.0029
P_{N2} (atm)	0.66	0.66	0.62	0.62	0.64	0.65
P_{Ar} (atm)	0.0079	0.0072	0.0072	0.0072	0.0072	0.0072
P_{CH4} (atm)	0.097	0.10	0.13	0.32	0.36	0.39
P_{O2} (atm)	0.036	0.010	0.0055	0.0043	0.0036	0.0029
P_{H2} (atm)	b.d.l.	b.d.l.	b.d.l.	0.00062	0.00062	0.00099
P_{He} (atm)	0.000005	0.000005	0.000006	0.000008	0.000005	0.000009
P_{TOT} (atm)	0.80	0.78	0.77	0.95	1.01	1.06

b.d.l. = below detection limit

Supplementary Table 5 Percentages of detected molecule that showed positive or negative significant correlation with CH_4 (Spearman correlation $p < 0.01$). Numbers in parenthesis describes the number of detected molecule in each molecular group.

	**Negative**	**Positive**
All molecule (2383)	10.1	11.7
O-poor Aliphatics (285)	0.3	25.0
O-rich Aliphatics (76)	5.5	35.6
O-poor unsaturated molecule (1040)	5.1	11.4
O-rich unsaturated molecule (636)	18.0	4.6
Aromatics & Condensed aromatics (207)	34.0	0.5
N-bearing molecule (934)	7.0	13.0

Supplementary Table 6 Total reads assigned to archaeal taxa (alphabetic order). Dominant genera are coded by letters and numbers (A1, A2, A3, etc.). Data are graphically visualized in figure 3 of the main text.

Taxon	Code	0.0m	0.5m	1.0m	2.0m	3.0m	4.5m	Sed0	Sed1
p_Altiarchaeota;c_Altiarchaeia	90	1786	1717	1559	1688	2364	1	0	0
p_Crenarchaeota;c_Bathyarchaeia	91	338	639	260	441	168	786	1656	1320
p_Crenarchaeota;c_Crenarchaeota Incertae Sedis;o_Aigarchaeales;f_Caldiarchaeaceae;g_Candidatus Caldiarchaeum	A14	0	0	27	0	0	0	0	0
p_Crenarchaeota;c_Crenarchaeota Incertae Sedis;o_Aigarchaeales;f_Geothermarchaeaceae	93	11	0	26	52	0	0	0	0
p_Crenarchaeota;c_Thermoprotei;o_Thermoproteales;f_Thermofilaceae;g_Thermofilum	A13	0	36	0	0	22	0	0	0
p_Crenarchaeota;c_Verstraetearchaeia;o_Methanomethyliales;f_Methanomethyliaceae;g_Candidatus Methanomethylicus	A12	0	0	0	34	68	0	0	0
p_Diapherotrites;c_Iainarchaeia	96	0	0	0	31	0	7	0	0
p_Diapherotrites;c_Micrarchaeia	97	14	0	0	0	0	39	3	0
p_Euryarchaeota;c_Halobacteria;o_Halobacteriales;f_Haloferacaceae;g_Natronococcus	A16	0	0	0	0	0	0	2	0
p_Euryarchaeota;c_Methanobacteria;o_Methanobacteriales	99	0	0	0	0	0	0	21	0
p_Euryarchaeota;c_Methanobacteria;o_Methanobacteriales;f_Methanobacteriaceae	100	8	0	0	0	0	0	74	0
p_Euryarchaeota;c_Methanobacteria;o_Methanobacteriales;f_Methanobacteriaceae;g_Methanobacterium	A1	295	91	197	135	50	29	7109	491
p_Euryarchaeota;c_Methanobacteria;o_Methanobacteriales;f_Methanobacteriaceae;g_Methanobrevibacter	A17	0	0	0	0	0	0	2	0
p_Euryarchaeota;c_Methanobacteria;o_Methanobacteriales;f_Methanothermobacteraceae;g_Methanothermobacter	A3	1752	1262	1844	732	1189	3	0	0
p_Euryarchaeota;c_Methanomicrobia	104	0	30	0	0	0	0	0	0
p_Euryarchaeota;c_Methanomicrobia;o_Methanocellales;f_Methanocellaceae;g_Methanocella	A15	0	0	0	0	0	0	0	20
p_Euryarchaeota;c_Methanomicrobia;o_Methanomicrobiales;f_Methanocorpusculaceae;g_Methanocalculus	A6	2	0	0	0	0	2520	68	1331
p_Euryarchaeota;c_Methanomicrobia;o_Methanomicrobiales;f_Methanomicrobiaceae	107	0	0	0	0	0	0	172	88
p_Euryarchaeota;c_Methanomicrobia;o_Methanomicrobiales;f_Methanomicrobiaceae;g_Methanoculleus	A2	1772	1150	1790	1191	1538	8	0	0
p_Euryarchaeota;c_Methanomicrobia;o_Methanomicrobiales;f_Methanoregulaceae	109	0	0	51	0	0	0	0	0
p_Euryarchaeota;c_Methanomicrobia;o_Methanomicrobiales;f_Methanoregulaceae;g_Methanolinea	A4	0	3	0	0	30	49	347	4496
p_Euryarchaeota;c_Methanomicrobia;o_Methanomicrobiales;f_Methanospirillaceae;g_Methanospirillum	A9	65	0	100	20	0	133	0	0
p_Euryarchaeota;c_Methanomicrobia;o_Methanosarcinales;f_Methanosaetaceae;g_Methanosaeta	A5	428	76	281	299	440	430	1071	1269
p_Euryarchaeota;c_Methanomicrobia;o_Methanosarcinales;f_Methanosarcinaceae;g_Methanosarcina	A8	255	210	474	129	240	0	46	0
p_Euryarchaeota;c_Thermococci;o_Methanofastidiosales	114	0	0	0	0	0	0	0	10
p_Euryarchaeota;c_Thermococci;o_Methanofastidiosales;f_Methanofastidiosaceae;g_Candidatus Methanofastidiosum	A7	264	167	120	195	20	1557	9	4
p_Euryarchaeota;c_Thermoplasmata	116	0	0	0	0	0	0	27	175
p_Euryarchaeota;c_Thermoplasmata;o_JdFR-43	117	0	33	0	23	0	0	0	0
p_Euryarchaeota;c_Thermoplasmata;o_Marine Benthic Group D and DHVEG-1	118	0	0	0	0	0	5	282	175
p_Euryarchaeota;c_Thermoplasmata;o_Methanomassiliicoccales	119	0	135	0	27	195	0	0	0
p_Euryarchaeota;c_Thermoplasmata;o_Methanomassiliicoccales;f_Methanomassiliicoccaceae	120	69	77	0	0	0	0	121	897
p_Euryarchaeota;c_Thermoplasmata;o_Methanomassiliicoccales;f_Methanomethylophilaceae	121	71	23	0	0	0	32	8	30
p_Euryarchaeota;c_Thermoplasmata;o_Methanomassiliicoccales;f_Methanomethylophilaceae;g_Candidatus Methanogranum	A11	0	172	0	0	0	0	0	0
p_Hadesarchaeaeota	123	0	0	4	0	0	0	32	490
p_Korarchaeota;c_Korarchaeia;o_Korarchaeales;f_Korarchaeaceae;g_Candidatus Korarchaeum	A10	82	50	70	49	47	0	0	0
p_Nanoarchaeaeota;c_Woesearchaeia	125	191	471	270	825	291	111	14	113
p_Nanoarchaeaeota;c_Woesearchaeia;o_Candidatus Staskawiczbacteria bacterium	126	43	134	95	250	131	1	0	0
p_Thaumarchaeota;c_Nitrososphaeria;o_Nitrososphaerales;f_Nitrososphaeraceae	127	0	0	0	0	0	0	0	7
p_Thaumarchaeota;c_Nitrososphaeria;o_Nitrososphaerales;f_Nitrososphaeraceae;g_Candidatus Nitrososphaera	A18	0	0	0	0	0	0	1	0

Supplementary Table 7 Total reads assigned to bacterial taxa (alphabetic order). Dominant genera are coded by letters and numbers (B1, B2, B3, etc.). Data are graphically visualized in figure 3 of the main text.

Taxon	Code	0.0m	0.5m	1.0m	2.0m	3.0m	4.0m	4.5m	Sed0	Sed1
p_Actinobacteria	1	1	0	4	0	0	2	0	0	13
p_Actinobacteria;c_Acidimicrobiia	2	78	143	159	133	102	4	0	0	0
p_Actinobacteria;c_Acidimicrobiia;o_IMCC26256	3	0	0	35	0	0	0	0	0	0
p_Actinobacteria;c_Acidimicrobiia;o_Microtrichales	4	205	331	418	234	280	137	0	0	0
p_Actinobacteria;c_Acidimicrobiia;o_Microtrichales;f_Ilumatobacteraceae	5	87	71	0	124	0	0	0	0	0
p_Actinobacteria;c_Acidimicrobiia;o_Microtrichales;f_Ilumatobacteraceae;g_CL500-29 marine group	B21	2	10	0	6	0	0	0	0	0
p_Actinobacteria;c_Acidimicrobiia;o_Microtrichales;f_Microtrichaceae;g_IMCC26207	B16	0	0	21	0	0	119	0	0	0
p_Actinobacteria;c_Actinobacteria	8	0	0	0	203	0	0	0	2	0
p_Actinobacteria;c_Actinobacteria;o_Micrococcales;f_Microbacteriaceae	9	0	0	1	0	0	0	0	0	0
p_Actinobacteria;c_Actinobacteria;o_Micrococcales;f_Microbacteriaceae;g_ML602J-51	B5	254	774	716	574	964	0	0	0	0
p_Actinobacteria;c_WCHB1-81	11	0	0	0	0	8	0	0	252	0
p_Armatimonadetes	12	0	0	0	0	0	0	0	46	0
p_Armatimonadetes;c_Chthonomonadetes;o_Chthonomonadales	13	0	0	0	0	0	0	0	236	0
p_Armatimonadetes;c_Fimbriimonadia;o_Fimbriimonadales;f_Fimbriimonadaceae	14	323	1971	3362	1149	2481	24	0	0	0
p_Bacteroidetes;c_Bacteroidia	15	0	0	0	0	0	4	13	1	10
p_Bacteroidetes;c_Bacteroidia;o_Bacteroidales	16	0	0	0	0	0	0	0	525	0
p_Bacteroidetes;c_Bacteroidia;o_Bacteroidales;f_ML635J-40 aquatic group	17	0	0	0	0	0	8313	12482	214	327
p_Bacteroidetes;c_Bacteroidia;o_Chitinophagales;f_Saprospiraceae	18	0	47	0	0	0	0	0	0	0
p_Bacteroidetes;c_Bacteroidia;o_Sphingobacteriales	19	0	0	0	0	0	0	6	0	0
p_Bacteroidetes;c_Bacteroidia;o_Sphingobacteriales;f_Lentimicrobiaceae	20	0	80	0	0	0	0	0	0	0
p_Bacteroidetes;c_Bacteroidia;o_Sphingobacteriales;f_NS11-12 marine group	21	0	10	0	0	0	0	0	0	0
p_Bacteroidetes;c_Ignavibacteria;o_Ignavibacteriales;f_BSN166	22	0	0	0	0	0	0	0	1058	0
p_Bacteroidetes;c_Rhodothermia;o_Balneolales;f_Balneolaceae;g_CK06-06-Mud-MAS4B-21	23	0	196	97	205	104	0	146	0	0
p_Bacteroidetes;c_Rhodothermia;o_Balneolales;f_Balneolaceae;g_F1-37X2	B11	0	0	0	0	0	333	0	0	0
p_Chloroflexi	25	0	0	0	0	0	0	0	42	0
p_Chloroflexi;c_Anaerolineae;o_Anaerolineales;f_Anaerolineaceae	26	0	0	0	0	0	0	0	0	119
p_Chloroflexi;c_Anaerolineae;o_Anaerolineales;f_Anaerolineaceae;g_ADurb.Bin120	B7	22	0	35	0	0	826	660	191	21
p_Chloroflexi;c_Dehalococcoidia;o_FS117-23B-02	28	0	0	0	0	0	0	0	62	36
p_Chloroflexi;c_Dehalococcoidia;o_GIF9	29	0	0	0	0	0	0	0	0	59
p_Chloroflexi;c_Dehalococcoidia;o_GIF9;f_AB-539-J10;g_SCGC-AB-539-J10	B2	0	0	0	0	0	0	664	9388	16469
p_Chloroflexi;c_Dehalococcoidia;o_MSBL5	31	0	0	0	0	0	0	0	53	30
p_Chloroflexi;c_Dehalococcoidia;o_vadinBA26	32	0	0	0	0	0	21	0	0	0
p_Cyanobacteria;c_Oxyphotobacteria	33	1360	574	488	594	160	0	0	0	0
p_Cyanobacteria;c_Oxyphotobacteria;o_Chloroplast	34	10	0	0	0	0	0	0	0	0
p_Cyanobacteria;c_Oxyphotobacteria;o_Nostocales	35	0	0	84	0	4	0	0	0	0
p_Cyanobacteria;c_Oxyphotobacteria;o_Nostocales;f_Microcystaceae	36	0	0	0	0	3	0	0	0	0
p_Cyanobacteria;c_Oxyphotobacteria;o_Nostocales;f_Microcystaceae;g_Synechocystis PCC-6803	B6	901	452	372	511	267	321	0	0	0
p_Cyanobacteria;c_Oxyphotobacteria;o_Nostocales;f_Nostocaceae;g_Nodularia PCC-9350	B14	0	0	0	0	0	0	0	206	0
p_Cyanobacteria;c_Oxyphotobacteria;o_Synechococcales;f_Cyanobiaceae	39	327	835	732	674	665	0	0	0	0
p_Cyanobacteria;c_Oxyphotobacteria;o_Synechococcales;f_Cyanobiaceae;g_Cyanobium PCC-6307	B1	9398	7042	8034	9122	9917	1968	431	0	0
p_Deinococcus-Thermus;c_Deinococci;o_Deinococcales;f_Trueperaceae;g_Truepera	B3	1959	1698	1182	1721	890	198	134	0	0
p_Firmicutes;c_Clostridia	42	0	0	0	0	0	4	0	3	0
p_Firmicutes;c_Clostridia;o_Clostridiales	43	0	0	0	0	0	0	55	2	0
p_Firmicutes;c_Clostridia;o_Clostridiales;f_Clostridiaceae 2;g_Alkaliphilus	B19	0	0	0	0	0	0	0	0	45
p_Firmicutes;c_Clostridia;o_Clostridiales;f_Ruminococcaceae	45	0	0	0	0	0	173	0	0	0
p_Firmicutes;c_Clostridia;o_Clostridiales;f_Syntrophomonadaceae	46	0	0	0	0	0	2309	156	4272	492
p_Firmicutes;c_Clostridia;o_Clostridiales;f_Syntrophomonadaceae;g_Dethiobacter	B4	0	20	0	0	0	2004	1629	833	100
p_Firmicutes;c_Clostridia;o_Thermoanaerobacterales;f_SRB2	48	0	0	16	0	0	0	0	0	0
p_Gemmatimonadetes;c_BD2-11 terrestrial group	49	0	0	0	0	0	0	0	1	0
p_Kiritimatiellaeota;c_Kiritimatiellae;o_WCHB1-41	50	0	0	0	0	0	0	45	0	0
p_Latescibacteria	51	0	0	0	0	0	0	0	41	0
p_Omnitrophicaeota	52	0	0	0	0	0	95	37	0	0
p_Patescibacteria;c_Berkelbacteria	53	0	0	0	0	0	14	0	0	0
p_Patescibacteria;c_Gracilibacteria;o_Absconditabacteriales (SR1)	54	0	61	31	37	37	0	0	0	0
p_Patescibacteria;c_Gracilibacteria;o_Candidatus Peregrinibacteria	55	0	0	0	0	0	0	202	0	0
p_Proteobacteria	56	136	154	137	138	102	0	33	0	0
p_Proteobacteria;c_Alphaproteobacteria	57	14	13	2	25	4	1	0	0	0
p_Proteobacteria;c_Alphaproteobacteria;o_Acetobacterales;f_Acetobacteraceae	58	0	0	0	25	0	0	0	0	0
p_Proteobacteria;c_Alphaproteobacteria;o_Caulobacterales;f_Hyphomonadaceae;g_Hyphomonas	B24	0	10	0	0	0	0	0	0	0
p_Proteobacteria;c_Alphaproteobacteria;o_Micavibrionales;f_Micavibrionaceae	60	0	39	20	22	0	0	0	0	0
p_Proteobacteria;c_Alphaproteobacteria;o_Rhizobiales;f_Rhizobiaceae	61	0	0	0	1	0	0	0	0	0
p_Proteobacteria;c_Alphaproteobacteria;o_Rhodobacterales;f_Rhodobacteraceae	62	545	432	622	202	200	1	0	0	19
p_Proteobacteria;c_Alphaproteobacteria;o_Rhodobacterales;f_Rhodobacteraceae;g_Halodurantibacterium	B25	0	0	3	0	0	0	0	0	0
p_Proteobacteria;c_Alphaproteobacteria;o_Rhodobacterales;f_Rhodobacteraceae;g_Rhodobacter	B22	3	3	5	0	0	0	0	0	0
p_Proteobacteria;c_Alphaproteobacteria;o_Rhodobacterales;f_Rhodobacteraceae;g_Rhodobaculum	B10	176	157	166	150	0	0	0	0	0
p_Proteobacteria;c_Alphaproteobacteria;o_Rhodobacterales;f_Rhodobacteraceae;g_Roseivivax	B8	443	337	184	360	213	90	0	0	0
p_Proteobacteria;c_Alphaproteobacteria;o_Rhodobacterales;f_Rhodobacteraceae;g_Rubribacterium	B23	0	0	0	0	0	11	0	0	0
p_Proteobacteria;c_Alphaproteobacteria;o_Rhodobacterales;f_Rhodobacteraceae;g_Tabrizicola	B26	0	2	0	0	0	0	0	0	0
p_Proteobacteria;c_Alphaproteobacteria;o_Rhodobacterales;f_Rhodobacteraceae;g_Tropicimonas	B20	16	10	0	0	0	0	0	0	0
p_Proteobacteria;c_Alphaproteobacteria;o_Rickettsiales;f_Mitochondria	70	0	0	0	0	0	0	0	0	22
p_Proteobacteria;c_Alphaproteobacteria;o_Sphingomonadales;f_Sphingomonadaceae	71	0	6	0	0	0	0	0	0	0
p_Proteobacteria;c_Alphaproteobacteria;o_Thalassobaculales	72	96	76	0	0	0	0	0	0	0
p_Proteobacteria;c_Deltaproteobacteria;o_Desulfuromonadales;f_Desulfuromonadaceae;g_Pelobacter	B13	0	0	0	0	0	0	0	269	0
p_Proteobacteria;c_Deltaproteobacteria;o_Oligoflexales;f_0319-6G20culture clone B126(2011)	74	0	11	14	0	0	0	0	0	0
p_Proteobacteria;c_Deltaproteobacteria;o_Syntrophobacterales;f_Syntrophaceae	75	0	0	0	0	0	229	204	0	0
p_Proteobacteria;c_Gammaproteobacteria	76	778	561	353	647	0	0	0	0	0
p_Proteobacteria;c_Gammaproteobacteria;o_Betaproteobacteriales	77	0	0	0	0	1	0	0	0	0
p_Proteobacteria;c_Gammaproteobacteria;o_Betaproteobacteriales;f_Burkholderiaceae;g_Hydrogenophaga	B18	28	0	28	0	13	0	0	0	0
p_Proteobacteria;c_Gammaproteobacteria;o_Betaproteobacteriales;f_Rhodocyclaceae;g_Azoarcus	B9	388	355	0	298	514	0	0	0	0
p_Proteobacteria;c_Gammaproteobacteria;o_Ectothiorhodospirales;f_Ectothiorhodospiraceae;g_Thioalkalivibrio	B17	0	93	0	0	0	0	0	0	0
p_Tenericutes;c_Mollicutes	81	0	17	1	0	19	0	0	0	0
p_Tenericutes;c_Mollicutes;o_EUB33-2	82	0	81	52	0	131	0	0	0	0
p_Tenericutes;c_Mollicutes;o_Izimaplasmatales;f_Izimaplasmataceae	83	0	330	170	205	308	0	0	0	0
p_Thermotogae;c_Thermotogae;o_Kosmotogales;f_Kosmotogaceae	84	0	0	0	0	0	43	0	0	0
p_Verrucomicrobia;c_Verrucomicrobiae;o_Chthoniobacterales;f_Terrimicrobiaceae;g_Terrimicrobium	B12	0	110	97	0	94	0	0	0	0
p_Verrucomicrobia;c_Verrucomicrobiae;o_Opitutales;f_Puniceicoccaceae	86	0	2	4	0	0	0	0	0	0
p_Verrucomicrobia;c_Verrucomicrobiae;o_Opitutales;f_Puniceicoccaceae;g_Puniceicoccus	B15	0	90	0	0	74	0	0	0	0
p_Verrucomicrobia;c_Verrucomicrobiae;o_Verrucomicrobiales;f_DEV007	88	0	0	15	0	0	0	0	0	0
p_WS1	89	0	0	0	0	0	0	0	80	0

Supplementary Table 8 Identifiers, taxonomic affiliation at genus level and relative abundance range (% of total reads) of the most prevalent cyanobacterial ASVs discussed in the study.

Code	ASV identifier	Genus	Range of relative abundance (% of total reads)
ASV1	0272907b5a3a9ce989dadef24e3edf07	*Synechocystis*	0.0-4.0
ASV2	0b0988ffcf44dd77b0f5a8c1c7438a26	*Synechocystis*	0.0-1.0
ASV3	39db866051011332c326d7abe187c004	*Synechocystis*	0.0-2.1
ASV4	6ffd7bca161c114512744e8470f5d38b	*Cyanobium*	0.4-5.8
ASV5	8d78c880a10ff50576c568cdcf390f81	*Cyanobium*	1.4-5.6
ASV6	d95df554c7eed94e55e9c8ea324cd706	*Cyanobium*	0.0-8.8
ASV7	210f3f7cef85bb5677d9c95000933c2c	*Cyanobium*	8.6-27.0

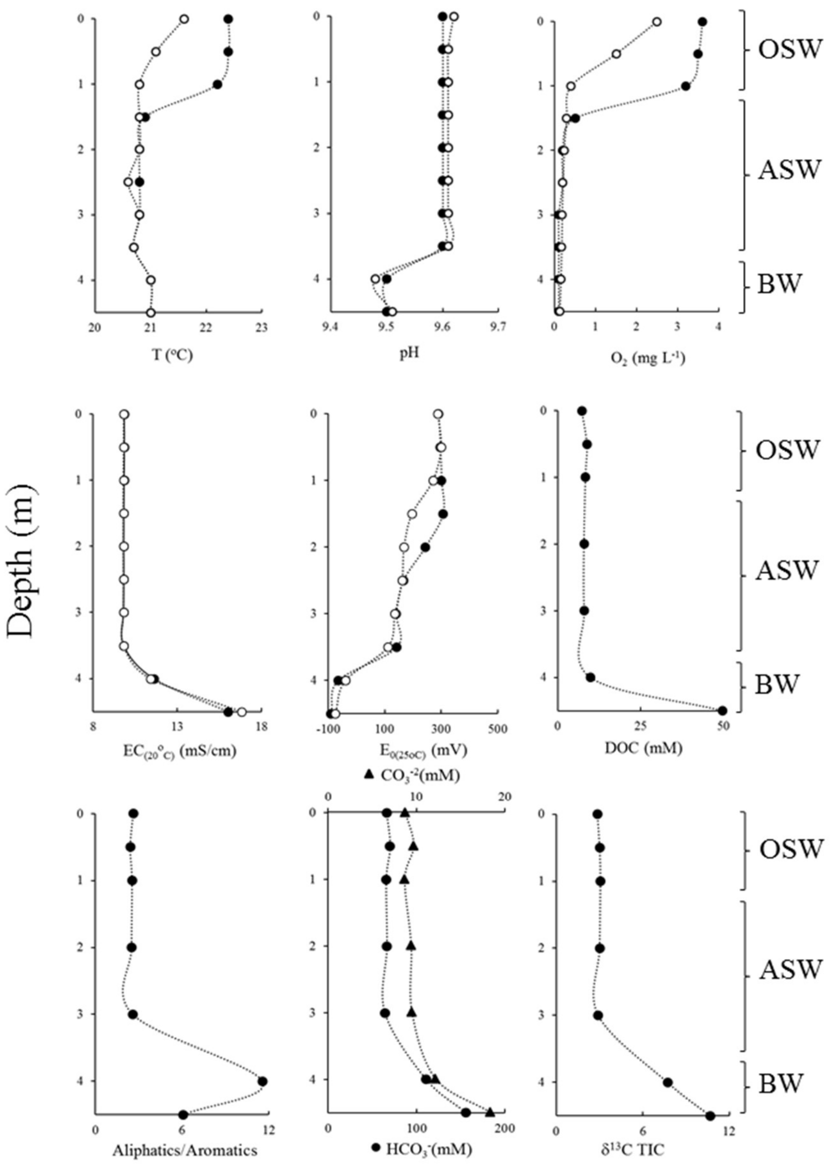

Supplementary Figure 3 Vertical profiles of water temperature, pH, dissolved O_2, electrical conductivity, redox, DOC, aliphatic:aromatic ratio, HCO_3^-, CO_3^{2-}, and $\delta^{13}C$-TDIC. The white and black dots refer to monitoring at 11:30 AM and at 03:15 PM, respectively.

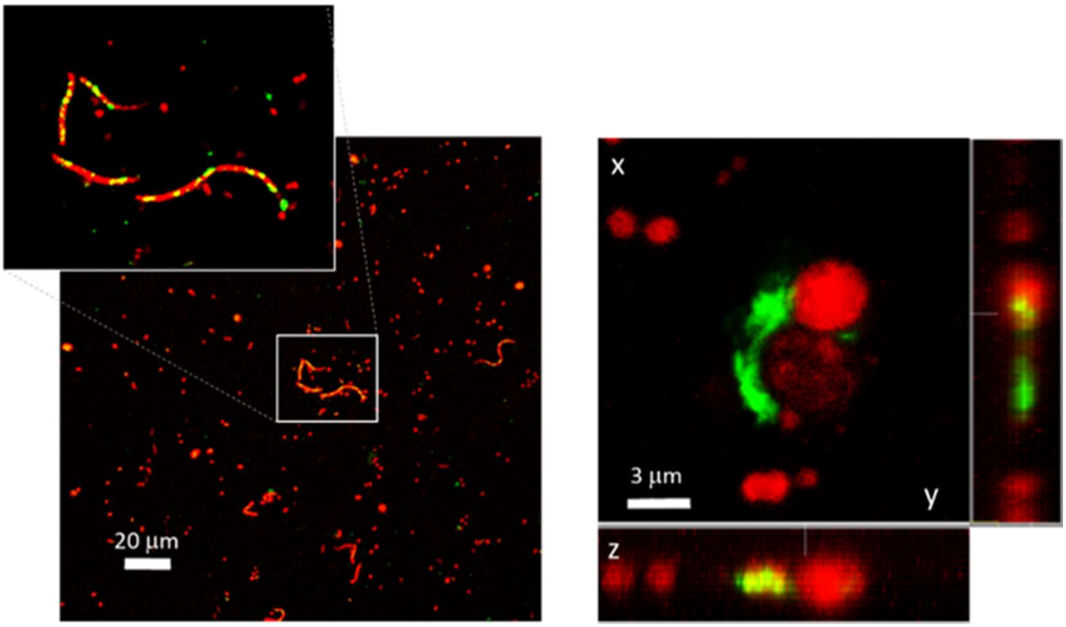

Supplementary Figure 4 CLSM combined images of Cyanobacteria (red) and Archaea cells (green) identified by CARD-FISH (left panel), and 3D spatial distribution (X−Y, X−Z, and Y−Z planes) of aggregated cells (right panel) in OSW at -0.5 m depth.

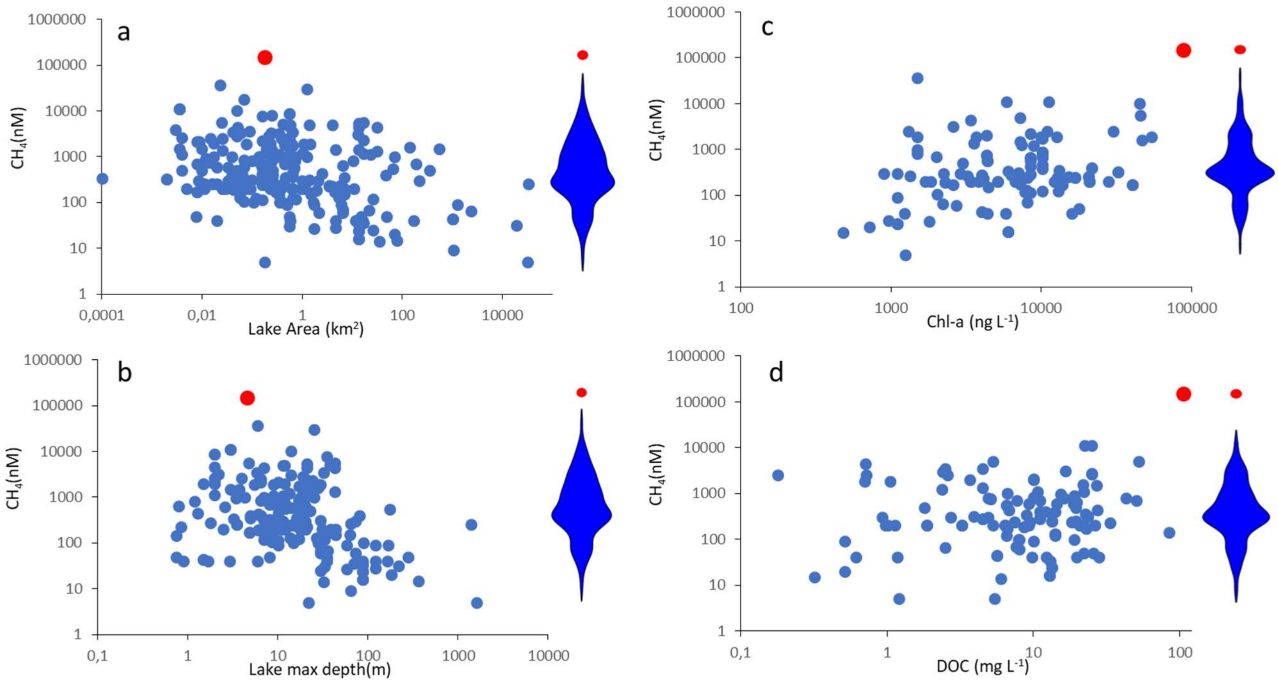

Supplementary Figure 5 Dissolved methane concentrations in oxic waters from lakes distributed worldwide. Plots show the relationship between CH_4 concentration, lake morphology (area, depth), and trophic status (Chl-a, DOC). Red dots indicates the values measured in waters from Lake Sonachi. All values are reported in the supplementary table 1. The bivariate relations in panels a, b, and c are statistically significant ($p < 0.01$).

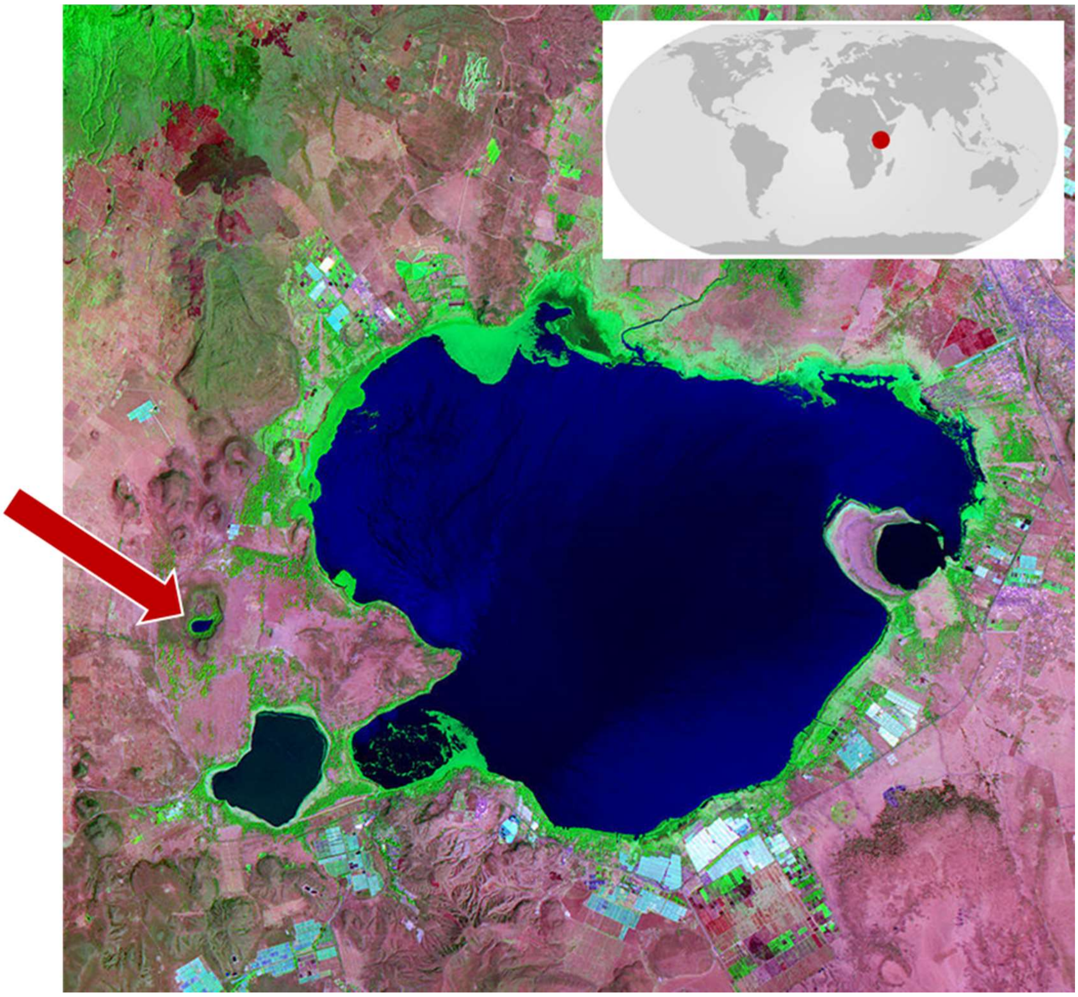

Supplementary Figure 6 Satellite image of Lake Naivasha, Kenya captured by NASA's Terra satellite on February 2, 2008 (http://earthobservatory.nasa.gov/IOTD/view.php?id=8599). Arrow indicates Lake Sonachi.

Supplementary references

1. Boehrer, B., Herzsprung, P., Schultze, M. & Millero, F. J. Calculating density of water in geochemical lake stratification models. *Limnol. Oceanogr. Methods* **8**, 567–574 (2010).
2. Allen, D. J., Darling, W. G. & Burgess, W. G. *Geothermics and hydrogeology of the southern part of the Kenya Rift Valley with emphasis on the Magadi-Nakuru area*. (British Geological Survey, 1989).
3. Becht, R., Mwango, F. & Muno, F. Groundwater links between Kenyan Rift Valley lakes. in *Climate Change 2013 - The Physical Science Basis* (ed. Intergovernmental Panel on Climate Change) 1–30 (Cambridge University Press, 2006). doi:10.1017/CBO9781107415324.004
4. Verschuren, D. Influence of depth and mixing regime on sedimentation in a small, fluctuating tropical soda lake. *Limnol. Oceanogr.* **44**, 1103–1113 (1999).
5. Odada, E. O. *Stable isotopic composition of East African lake waters*. (2001).
6. Craig, H. Isotopic Variations in Meteoric Waters. *Science (80-.).* **133**, 1702–1703 (1961).
7. Ojiambo, B. S., Poreda, R. J. & Lyons, W. B. Ground Water/Surface Water Interactions in Lake Naivasha, Kenya, Using δ18O, δD, and 3H/3He Age-Dating. *Ground Water* **39**, 526–533 (2001).
8. Sepulveda-Jauregui, A., Walter Anthony, K. M., Martinez-Cruz, K., Greene, S. & Thalasso, F. Methane and carbon dioxide emissions from 40 lakes along a north–south latitudinal transect in Alaska. *Biogeosciences* **12**, 3197–3223 (2015).
9. Roland, F. A. E., Darchambeau, F., Morana, C., Bouillon, S. & Borges, A. V. Emission and oxidation of methane in a meromictic, eutrophic and temperate lake (Dendre, Belgium). *Chemosphere* **168**, 756–764 (2017).
10. Rinta, P. *et al.* An inter-regional assessment of concentrations and δ13C values of methane and dissolved inorganic carbon in small European lakes. *Aquat. Sci.* **77**, 667–680 (2015).

11. Taipale, S., Kankaala, P., Hahn, M., Jones, R. & Tiirola, M. Methane-oxidizing and photoautotrophic bacteria are major producers in a humic lake with a large anoxic hypolimnion. *Aquat. Microb. Ecol.* **64**, 81–95 (2011).

12. Lambrecht, N. *et al.* Biogeochemical and physical controls on methane fluxes from two ferruginous meromictic lakes. *Geobiology* **18**, 54–69 (2020).

13. Encinas Fernández, J., Peeters, F. & Hofmann, H. On the methane paradox: Transport from shallow water zones rather than in situ methanogenesis is the major source of CH 4 in the open surface water of lakes. *J. Geophys. Res. Biogeosciences* **121**, 2717–2726 (2016).

14. Schilder, J., Bastviken, D., van Hardenbroek, M. & Heiri, O. Spatiotemporal patterns in methane flux and gas transfer velocity at low wind speeds: Implications for upscaling studies on small lakes. *J. Geophys. Res. Biogeosciences* **121**, 1456–1467 (2016).

15. Cabassi, J. *et al.* Biogeochemical processes involving dissolved CO2 and CH4 at Albano, Averno, and Monticchio meromictic volcanic lakes (Central–Southern Italy). *Bull. Volcanol.* **75**, 683 (2013).

16. Bédard, C. & Knowles, R. Some properties of methane oxidation in a thermally stratified lake. *Can. J. Fish. Aquat. Sci.* **54**, 1639–1645 (1997).

17. West, W. E., Coloso, J. J. & Jones, S. E. Effects of algal and terrestrial carbon on methane production rates and methanogen community structure in a temperate lake sediment. *Freshw. Biol.* **57**, 949–955 (2012).

18. Hamilton, J. D., Kelly, C. A., Rudd, J. W. M., Hesslein, R. H. & Roulet, N. T. Flux to the atmosphere of CH 4 and CO 2 from wetland ponds on the Hudson Bay lowlands (HBLs). *J. Geophys. Res.* **99**, 1495 (1994).

19. Striegl, R. G. & Michmerhuizen, C. M. Hydrologic influence on methane and carbon dioxide dynamics at two north-central Minnesota lakes. *Limnol. Oceanogr.* **43**, 1519–1529 (1998).

20. Martinez-Cruz, K., Sepulveda-Jauregui, A., Walter Anthony, K. & Thalasso, F. Geographic and seasonal variation of dissolved methane and aerobic methane oxidation in Alaskan lakes. *Biogeosciences* **12**, 4595–4606 (2015).

21. Kankaala, P., Huotari, J., Tulonen, T. & Ojala, A. Lake-size dependent physical forcing drives carbon dioxide and methane effluxes from lakes in a boreal landscape. *Limnol. Oceanogr.* **58**, 1915–1930 (2013).

22. Northington, R. M. & Saros, J. E. Factors Controlling Methane in Arctic Lakes of Southwest Greenland. *PLoS One* **11**, e0159642 (2016).

23. Repo, E. *et al.* Release of CO 2 and CH 4 from small wetland lakes in western Siberia. *Tellus B Chem. Phys. Meteorol.* **59**, 788–796 (2007).

24. Bastviken, D., Cole, J. J., Pace, M. L. & Van de Bogert, M. C. Fates of methane from different lake habitats: Connecting whole-lake budgets and CH 4 emissions. *J. Geophys. Res. Biogeosciences* **113**, 1–13 (2008).

25. Casper, P., Maberly, S. C., Hall, G. H. & Finlay, B. J. Fluxes of methane and carbon dioxide from a small productive lake to the atmosphere. *Biogeochemistry* **49**, 1–19 (2000).

26. Hofmann, H., Federwisch, L. & Peeters, F. Wave-induced release of methane: Littoral zones as source of methane in lakes. *Limnol. Oceanogr.* **55**, 1990–2000 (2010).

27. Vachon, D., Langenegger, T., Donis, D., Beaubien, S. E. & McGinnis, D. F. Methane emission offsets carbon dioxide uptake in a small productive lake. *Limnol. Oceanogr. Lett.* **5**, 384–392 (2020).

28. Grossart, H.-P., Frindte, K., Dziallas, C., Eckert, W. & Tang, K. W. Microbial methane production in oxygenated water column of an oligotrophic lake. *Proc. Natl. Acad. Sci.* **108**, 19657–19661 (2011).

29. Denfeld, B. A. *et al.* Constraints on methane oxidation in ice-covered boreal lakes. *J.*

Geophys. Res. Biogeosciences **121**, 1924–1933 (2016).

30. Thottathil, S. D., Reis, P. C. J. & Prairie, Y. T. Methane oxidation kinetics in northern freshwater lakes. *Biogeochemistry* **143**, 105–116 (2019).

31. Savvichev, A. S. *et al.* Microbial Processes and Microbial Communities in the Water Column of the Polar Meromictic Lake Bol'shie Khruslomeny at the White Sea Coast. *Front. Microbiol.* **11**, (2020).

32. Milucka, J. *et al.* Methane oxidation coupled to oxygenic photosynthesis in anoxic waters. *ISME J.* **9**, 1991–2002 (2015).

33. Barbosa, P. M. *et al.* High rates of methane oxidation in an Amazon floodplain lake. *Biogeochemistry* **137**, 351–365 (2018).

34. Huotari, J. *Carbon dioxide and methane exchange between a boreal pristine lake and the atmosphere*. (Helsingin Yliopisto, 2011).

35. Natchimuthu, S. *et al.* Spatio-temporal variability of lake CH 4 fluxes and its influence on annual whole lake emission estimates. *Limnol. Oceanogr.* **61**, S13–S26 (2016).

36. Donis, D. *et al.* Full-scale evaluation of methane production under oxic conditions in a mesotrophic lake. *Nat. Commun.* **8**, 1661 (2017).

37. DelSontro, T., del Giorgio, P. A. & Prairie, Y. T. No Longer a Paradox: The Interaction Between Physical Transport and Biological Processes Explains the Spatial Distribution of Surface Water Methane Within and Across Lakes. *Ecosystems* **21**, 1073–1087 (2018).

38. West, W. E., Creamer, K. P. & Jones, S. E. Productivity and depth regulate lake contributions to atmospheric methane. *Limnol. Oceanogr.* **61**, S51–S61 (2016).

39. Miller, L. G. & Oremland, R. S. Methane efflux from the pelagic regions of four lakes. *Global Biogeochem. Cycles* **2**, 269–277 (1988).

40. Pokrovsky, O. S., Shirokova, L. S., Kirpotin, S. N., Kulizhsky, S. P. & Vorobiev, S. N.

Impact of western Siberia heat wave 2012 on greenhouse gases and trace metal concentration in thaw lakes of discontinuous permafrost zone. *Biogeosciences* **10**, 5349–5365 (2013).

41. Zhang, L. *et al.* Spatial variations in diffusive methane fluxes and the role of eutrophication in a subtropical shallow lake. *Sci. Total Environ.* **759**, 143495 (2021).

42. Vagle, S., Hume, J., McLaughlin, F., MacIsaac, E. & Shortreed, K. A methane bubble curtain in meromictic Sakinaw Lake, British Columbia. *Limnol. Oceanogr.* **55**, 1313–1326 (2010).

43. Khatun, S. *et al.* Aerobic methane production by planktonic microbes in lakes. *Sci. Total Environ.* **696**, 133916 (2019).

44. Huttunen, J. T. *et al.* Fluxes of methane, carbon dioxide and nitrous oxide in boreal lakes and potential anthropogenic effects on the aquatic greenhouse gas emissions. *Chemosphere* **52**, 609–621 (2003).

45. Shirokova, L. S. *et al.* Biogeochemistry of organic carbon, CO2, CH4, and trace elements in thermokarst water bodies in discontinuous permafrost zones of Western Siberia. *Biogeochemistry* **113**, 573–593 (2013).

46. Khatun, S. *et al.* Linking Stoichiometric Organic Carbon–Nitrogen Relationships to planktonic Cyanobacteria and Subsurface Methane Maximum in Deep Freshwater Lakes. *Water* **12**, 402 (2020).

47. Utsumi, M. *et al.* Dynamics of dissolved methane and methane oxidation in dimictic Lake Nojiri during winter. *Limnol. Oceanogr.* **43**, 10–17 (1998).

48. Rudd, J. W. M. Methane oxidation in Lake Tanganyika (East Africa). *Limnol. Oceanogr.* **25**, 958–963 (1980).

49. Riera, J. L., Schindler, J. E. & Kratz, T. K. Seasonal dynamics of carbon dioxide and methane in two clear-water lakes and two bog lakes in northern Wisconsin, U.S.A. *Can. J. Fish. Aquat. Sci.* **56**, 265–274 (1999).

50. Linnaluoma, J. *Factors controlling carbon gas fluxes in boreal lakes*. (Helsingin yliopisto, 2012).

51. Lopes, F. *et al.* Biogeochemical modelling of anaerobic vs. aerobic methane oxidation in a meromictic crater lake (Lake Pavin, France). *Appl. Geochemistry* **26**, 1919–1932 (2011).

52. Borges, A. V., Abril, G., Delille, B., Descy, J.-P. & Darchambeau, F. Diffusive methane emissions to the atmosphere from Lake Kivu (Eastern Africa). *J. Geophys. Res.* **116**, G03032 (2011).

53. Blees, J. *et al.* Spatial variations in surface water methane super-saturation and emission in Lake Lugano, southern Switzerland. *Aquat. Sci.* **77**, 535–545 (2015).

54. Tassi, F. *et al.* The biogeochemical vertical structure renders a meromictic volcanic lake a trap for geogenic CO2 (Lake Averno, Italy). *PLoS One* **13**, e0193914 (2018).

55. Ray, A. K. Biogeochemical fluxes of carbon and nitrogen from Chilika lake East Coast of India. (Chennai, 2013).

56. Larmola, T. Contribution of vegetated littoral zone to winter fluxes of carbon dioxide and methane from boreal lakes. *J. Geophys. Res.* **109**, D19102 (2004).

57. López Bellido, J., Tulonen, T., Kankaala, P. & Ojala, A. CO 2 and CH 4 fluxes during spring and autumn mixing periods in a boreal lake (Pääjärvi, southern Finland). *J. Geophys. Res.* **114**, G04007 (2009).

58. Schmid, M. *et al.* Sources and sinks of methane in Lake Baikal: A synthesis of measurements and modeling. *Limnol. Oceanogr.* **52**, 1824–1837 (2007).

59. Butturini, A. *et al.* Dissolved organic matter in a tropical saline-alkaline lake of the East African Rift Valley. *Water Res.* **173**, 115532 (2020).
