# Supplementary material for: High concentrations of dissolved biogenic methane associated with cyanobacterial blooms in East African lake surface water
Source: Commun Biol. 2021 Jul 7;4:845. doi: 10.1038/s42003-021-02365-x (PMC8263762; doi:10.1038/s42003-021-02365-x)
Supplement: Supplementary file 2 — Reporting Summary [file 42003_2021_2365_MOESM2_ESM.pdf]

nature research

Corresponding author(s): Stefano Fazi

Last updated by author(s): May 21, 2021

Reporting Summary

Nature Research wishes to improve the reproducibility of the work that we publish. This form provides structure for consistency and transparency in reporting. For further information on Nature Research policies, see our Editorial Policies and the Editorial Policy Checklist.

Statistics

For all statistical analyses, confirm that the following items are present in the figure legend, table legend, main text, or Methods section.

n/a	Confirmed	
[ ]	[x]	The exact sample size (*n*) for each experimental group/condition, given as a discrete number and unit of measurement
[ ]	[x]	A statement on whether measurements were taken from distinct samples or whether the same sample was measured repeatedly
[ ]	[x]	The statistical test(s) used AND whether they are one- or two-sided<br>*Only common tests should be described solely by name; describe more complex techniques in the Methods section.*
[x]	[ ]	A description of all covariates tested
[ ]	[x]	A description of any assumptions or corrections, such as tests of normality and adjustment for multiple comparisons
[ ]	[x]	A full description of the statistical parameters including central tendency (e.g. means) or other basic estimates (e.g. regression coefficient) AND variation (e.g. standard deviation) or associated estimates of uncertainty (e.g. confidence intervals)
[x]	[ ]	For null hypothesis testing, the test statistic (e.g. *F*, *t*, *r*) with confidence intervals, effect sizes, degrees of freedom and *P* value noted<br>*Give P values as exact values whenever suitable.*
[x]	[ ]	For Bayesian analysis, information on the choice of priors and Markov chain Monte Carlo settings
[x]	[ ]	For hierarchical and complex designs, identification of the appropriate level for tests and full reporting of outcomes
[x]	[ ]	Estimates of effect sizes (e.g. Cohen's *d*, Pearson's *r*), indicating how they were calculated

Our web collection on statistics for biologists contains articles on many of the points above.

Software and code

Policy information about availability of computer code

Data collection	No software and code were used for data collection in this study
Data analysis	Data elaborations were computed using the software PAST (version 4.0)

For manuscripts utilizing custom algorithms or software that are central to the research but not yet described in published literature, software must be made available to editors and reviewers. We strongly encourage code deposition in a community repository (e.g. GitHub). See the Nature Research guidelines for submitting code & software for further information.

Data

Policy information about availability of data

All manuscripts must include a data availability statement. This statement should provide the following information, where applicable:

- Accession codes, unique identifiers, or web links for publicly available datasets
- A list of figures that have associated raw data
- A description of any restrictions on data availability

Authors can confirm that all relevant data are included in the paper and/or its supplementary information files

Field-specific reporting

Ecological, evolutionary & environmental sciences study design

All studies must disclose on these points even when the disclosure is negative.

Study description	Water and dissolved gas sampling was carried out along the vertical profile from surface to bottom (at 0, 0.5, 1, 2, 3, 4 and 4.5 m depth) using the single hose method at the central area of the Sonachi lake. Sediments were collected by a crab. All data were quantitative and were obtained as described in the method section. The only semi-quantitative data were those obtained by amplicon sequencing.
Research sample	The rationale of sample choice was guided by the need to depict water column stratification, as a compromise of the number of samples that could be properly collected, handled and transported from such a remote area. Based on a preliminary profile of physicochemical characteristics (by multiple probe) we defined the sampling depth, based on the water column stratification. Another challenge for the number of samples, was related to the possibility to collect aliquots for dissolved gases analysis by a wooden artisanal raft, submerging a Rilsan® tube to the desired depth and purging it through a syringe.
Sampling strategy	Aliquots of water were defined based on current analytical methods for chemical and microbiological analysis. The amount of water filtered for DNA extraction was until filter clogging.
Data collection	Data collection procedure is detailed in the method section. Sampling campaign was carried out by S. Fazi, S. Venturi, N. Pacini, E. Vazquez, Lydia A. Olaka and A. Butturini, with the kind logistic support of Mr. Silas W. Wanjala of the Naivasha Riparian Association and Mr. Lawi Kiplimo, Head Manager of the Crater Lake Sanctuary. During sampling campaign, the physical-chemical parameters were recorded in situ by a probe. Samples were fixed, filtered, stored at the Lake Naivasha Riparian Association Camp, were a field laboratory was available on the shore of the nearby Lake Naivasha. After that, aliquots were distributed among the different institutions that carried out the different analysis.
Timing and spatial scale	Samples were collected in one day at the spatial scale of meters
Data exclusions	No data were excluded
Reproducibility	No attempt to repeat the data collection was done, except for oxygen measurements that was repeated twice at 11.30 AM and 3.15 PM. The different results were interpreted as impact of primary production.
Randomization	Being collected along a vertical profile, our data were not randomised. We grouped the sampling point by depth.
Blinding	The extent of blinding during sampling depended on the fact that we defined the sampling depth a priori, based on the water column stratification. Moreover, during data acquisition samples were identified by numbers and the operators did not have direct access to correspondence with sample name.

Did the study involve field work? ☒ Yes ☐ No

Field work, collection and transport

Field conditions	As described in the method section, local climate at Lake Sonachi is warm and semiarid, with evaporation exceeding precipitation on an annual basis. Protection from wind by steep crater walls (rising up from 30 to 115 m above the lake surface) and vegetation (mainly Vachellia xanthophloea) limit water mixing. The hydrological balance is maintained by precipitation (~680 mm/year in the crater catchment) and evaporation (~1,870 mm/year). Furthermore, the occurrence of subsurface inflow from the near Lake Naivasha was proposed according to synchronous lake-level changes among the two lakes and other hydrological evidences. Chemical stratification and meromixis were documented across 8 years of periodic measurements and attributed to several local factors, including basin morphometry, diurnal periodicity of winds and thermal stratification, seasonal/yearly rainfall variations, and biological decomposition.
Location	Lake Sonachi is located at about 90 km NW of Nairobi at 1,884 m a.s.l., within the Eastern Rift Valley in central Kenya (0°46’57.68’’S; 36°16’’E).
Access & import/export	The study was performed under research clearance permit NACOSTI/P/16/23342/10489 Biodiversity studies in Kenya’s Rift Valley, granted to David M. Harper by the Government of Kenya. The study was conceived during the 2nd African International Symposium and Advanced Training Course on Ecohydrology for Water, Biodiversity, Ecosystem Services and Resilience in Africa November 2016 - organized by the UNESCO Ecohydrology Programme. Lake Naivasha basin is a demosite for the implementation of the UNESCO Ecohydrology Programme (Ecohydrology as the framework for sustainable utilization of water in the Naivasha basin - Kenya). During that symposium the study was conceived and discussed among different authors and Mr. Silas W. Wanjala of the Lake Naivasha Riparian Association. Moreover, Dr. Lydia A. Olaka from the Department of Geology (University of Nairobi, Kenya) joined the international team during the sampling campaign, collaborating on exploring hot springs adjacent to Lake Sonachi.
Disturbance	No disturbances need to be reported.

Reporting for specific materials, systems and methods

We require information from authors about some types of materials, experimental systems and methods used in many studies. Here, indicate whether each material, system or method listed is relevant to your study. If you are not sure if a list item applies to your research, read the appropriate section before selecting a response.

Materials & experimental systems

n/a	Involved in the study
☐	☐ Antibodies
☐	☐ Eukaryotic cell lines
☐	☐ Palaeontology and archaeology
☐	☐ Animals and other organisms
☐	☐ Human research participants
☐	☐ Clinical data
☐	☐ Dual use research of concern

Methods

n/a	Involved in the study
☐	☐ ChIP-seq
☐	☒ Flow cytometry
☐	☐ MRI-based neuroimaging

Antibodies

Antibodies used: n/a

Validation: n/a

Eukaryotic cell lines

Policy information about cell lines

Cell line source(s): n/a

Authentication: n/a

Mycoplasma contamination: n/a

Commonly misidentified lines (See ICLAC register): *Name any commonly misidentified cell lines used in the study and provide a rationale for their use.*

Palaeontology and Archaeology

Specimen provenance: n/a

Specimen deposition: n/a

Dating methods: n/a

☐ Tick this box to confirm that the raw and calibrated dates are available in the paper or in Supplementary Information.

Ethics oversight: n/a

Note that full information on the approval of the study protocol must also be provided in the manuscript.

Animals and other organisms

Policy information about studies involving animals; ARRIVE guidelines recommended for reporting animal research

Laboratory animals: n/a

Wild animals: n/a

Field-collected samples: n/a

Ethics oversight: n/a

Note that full information on the approval of the study protocol must also be provided in the manuscript.

Human research participants

Policy information about studies involving human research participants

Population characteristics: n/a

Recruitment: n/a

Ethics oversight: n/a

Note that full information on the approval of the study protocol must also be provided in the manuscript.

Clinical data

Policy information about clinical studies

All manuscripts should comply with the ICMJE guidelines for publication of clinical research and a completed CONSORT checklist must be included with all submissions.

Clinical trial registration	n/a
Study protocol	n/a
Data collection	n/a
Outcomes	n/a

Dual use research of concern

Policy information about dual use research of concern

Hazards

Could the accidental, deliberate or reckless misuse of agents or technologies generated in the work, or the application of information presented in the manuscript, pose a threat to:

No	Yes	
☐	☐	Public health
☐	☐	National security
☐	☐	Crops and/or livestock
☐	☒	Ecosystems
☐	☐	Any other significant area

Experiments of concern

Does the work involve any of these experiments of concern:

No	Yes	
☒	☐	Demonstrate how to render a vaccine ineffective
☒	☐	Confer resistance to therapeutically useful antibiotics or antiviral agents
☒	☐	Enhance the virulence of a pathogen or render a nonpathogen virulent
☒	☐	Increase transmissibility of a pathogen
☒	☐	Alter the host range of a pathogen
☒	☐	Enable evasion of diagnostic/detection modalities
☒	☐	Enable the weaponization of a biological agent or toxin
☒	☐	Any other potentially harmful combination of experiments and agents

ChIP-seq

Data deposition

☐ Confirm that both raw and final processed data have been deposited in a public database such as GEO.

☐ Confirm that you have deposited or provided access to graph files (e.g. BED files) for the called peaks.

Data access links<br>*May remain private before publication.*	n/a
Files in database submission	n/a
Genome browser session<br>(e.g. UCSC)	*Provide a link to an anonymized genome browser session for "Initial submission" and "Revised version" documents only, to enable peer review. Write "no longer applicable" for "Final submission" documents.*

Methodology

Replicates	n/a

Sequencing depth	n/a
Antibodies	n/a
Peak calling parameters	n/a
Data quality	n/a
Software	n/a

Flow Cytometry

Plots

Confirm that:

- [x] The axis labels state the marker and fluorochrome used (e.g. CD4-FITC).
- [x] The axis scales are clearly visible. Include numbers along axes only for bottom left plot of group (a 'group' is an analysis of identical markers).
- [x] All plots are contour plots with outliers or pseudocolor plots.
- [x] A numerical value for number of cells or percentage (with statistics) is provided.

Methodology

Sample preparation	Unfiltered and GFF-filtered water samples (2 mL) were fixed with a formaldehyde solution (final concentration 1%) and stored at 4°C until the analyses.
Instrument	A50-micro from Apogee Flow Systems
Software	Apogee Histogram (v89.0 - Apogee Flow System)
Cell population abundance	Absolute volumetric counts were performed by staining with SYBR Green I (1:10000 dilution). A threshold was set to the green channel and samples were run at low flow rate (< 1000 events per s-1).
Gating strategy	Fixed gates were designed to discriminate between free-living cells and aggregates according to their signatures in a side scatter vs. green fluorescence plot. Microbial aggregates were back-gated on a forward scatter histogram plot and divided into putative submicrometric and micrometric particles, respectively showing forward scatter signal intensities lower and higher than that of 1-µm size calibration beads used as internal standard. The .fcs files will be freely available at the Flow Repository identifier: https://flowrepository.org/id/[...].

- [x] Tick this box to confirm that a figure exemplifying the gating strategy is provided in the Supplementary Information.

Magnetic resonance imaging

Experimental design

Design type	n/a
Design specifications	n/a
Behavioral performance measures	n/a

Acquisition

Imaging type(s)	n/a
Field strength	n/a
Sequence & imaging parameters	n/a
Area of acquisition	n/a

Diffusion MRI ☐ Used ☐ Not used

Preprocessing

Preprocessing software	n/a

Normalization	n/a
Normalization template	n/a
Noise and artifact removal	n/a
Volume censoring	n/a

Statistical modeling & inference

Model type and settings	n/a
Effect(s) tested	n/a

Specify type of analysis: ☐ Whole brain ☐ ROI-based ☐ Both

Statistic type for inference (See Eklund et al. 2016)	*Specify voxel-wise or cluster-wise and report all relevant parameters for cluster-wise methods.*
Correction	n/a

Models & analysis

n/a	Involved in the study
☒	☐ Functional and/or effective connectivity
☒	☐ Graph analysis
☒	☐ Multivariate modeling or predictive analysis
